# (Semi)automated approaches to data extraction for systematic reviews and meta-analyses in social sciences: A living review

**DOI:** 10.12688/f1000research.151493.1

**Published:** 2024-06-20

**Authors:** Amanda Legate, Kim Nimon, Ashlee Noblin

**Affiliations:** 1 Human Resource Development, The University of Texas at Tyler, Tyler, Texas, 75799, USA

**Keywords:** Automated data extraction, systematic review, meta-analysis, evidence synthesis, social science research, APA Journal Article Reporting Standards (JARS)

## Abstract

**Background:**

An abundance of rapidly accumulating scientific evidence presents novel opportunities for researchers and practitioners alike, yet such advantages are often overshadowed by resource demands associated with finding and aggregating a continually expanding body of scientific information. Data extraction activities associated with evidence synthesis have been described as time-consuming to the point of critically limiting the usefulness of research. Across social science disciplines, the use of automation technologies for timely and accurate knowledge synthesis can enhance research translation value, better inform key policy development, and expand the current understanding of human interactions, organizations, and systems. Ongoing developments surrounding automation are highly concentrated in research for evidence-based medicine with limited evidence surrounding tools and techniques applied outside of the clinical research community. The goal of the present study is to extend the automation knowledge base by synthesizing current trends in the application of extraction technologies of key data elements of interest for social scientists.

**Methods:**

We report the baseline results of a living systematic review of automated data extraction techniques supporting systematic reviews and meta-analyses in the social sciences. This review follows PRISMA standards for reporting systematic reviews.

**Results:**

The baseline review of social science research yielded 23 relevant studies.

**Conclusions:**

When considering the process of automating systematic review and meta-analysis information extraction, social science research falls short as compared to clinical research that focuses on automatic processing of information related to the PICO framework. With a few exceptions, most tools were either in the infancy stage and not accessible to applied researchers, were domain specific, or required substantial manual coding of articles before automation could occur. Additionally, few solutions considered extraction of data from tables which is where key data elements reside that social and behavioral scientists analyze.

## Introduction

Across disciplines, systematic reviews and meta-analyses are integral to exploring and explaining phenomena, discovering causal inferences, and supporting evidence-based decision making. The concept of metascience represents an array of evidence synthesis approaches which support combining existing research results to summarize what is known about a specific topic ( Davis et al., 2014; Gough et al., 2020). Researchers use a variety of systematic review methodologies to synthesize evidence within their domains or to integrate extant knowledge bases spanning multiple disciplines and contexts. When engaging in quantitative evidence synthesis, researchers often supplement the systematic review with meta-analysis (a principled statistical process for grouping and summarizing quantitative information reported across studies within a research domain). As technology advances, in addition to greater access to data, researchers are presented with new forms and sources of data to support evidence synthesis ( Bosco et al. *,* 2017; Ip et al., 2012; Wagner et al., 2022).

Systematic reviews and meta-analyses are fundamental to supporting reproducibility and generalizability of research surrounding social and cultural aspects of human behavior, however, the process of extracting data from primary research is a labor-intensive effort, fraught with the potential for human error (see Pigott & Polanin, 2020; Yu et al., 2018). Comprehensive data extraction activities associated with evidence synthesis have been described as time-consuming to the point of critically limiting the usefulness of existing approaches ( Holub et al., 2021). Moreover, research indicates that it can take several years for original studies to be included in a new review due to the rapid pace of new evidence generation ( Jonnalagadda et al., 2015).

### The need for this review

In the clinical research domain, particularly in Randomized Control Trials (RCTs), automation technologies for data extraction are evolving rapidly (see Schmidt et al., 2023). In contrast with the more defined standards that have evolved throughout clinical research domains, within and across social sciences, substantial variation exists in research designs, reporting protocols, and even publication outlet standards ( Davis et al., 2014; Short et al., 2018; Wagner et al., 2022). In health intervention research, targeted data elements generally include Population (or Problem), Intervention, Control, and Outcome (i.e., PICO; see Eriksen and Frandsen, 2018; Tsafnat et al., 2014). While experimental designs are considered a gold-standard for translational value, many phenomena examined across the social sciences occur within contexts which necessitate research pragmatism in both design and methodological considerations ( Davis et al., 2014).

Consider, for example, the field of Human Resource Development (HRD). In HRD, a primary focal hub for research includes outcomes of workplace interventions intended to inform and improve areas such as learning, training, organizational development, and performance improvement ( Shirmohammadi et al., 2021). While measuring intervention outcomes is a substantial area of discourse, HRD researchers have predominantly relied on cross-sectional survey data and the most commonly employed quantitative method is Structural Equation Modeling ( Park et al., 2021). Thus, meta-analyses are increasingly essential for supporting reproducibility and generalizability of research. In these fields, data elements targeted for extraction would rarely align with the PICO framework, but rather, meta-analytic endeavors would entail extraction of measures such as effect sizes, model fit indices, or instrument psychometric properties ( Appelbaum et al., 2018).

### Related research

Serving as a model for the present study, Schmidt et al. (2023) are conducting a living systematic review (LSR) of tools and techniques available for (semi)automated extraction of data elements pertinent to synthesizing the effects of healthcare interventions (see Higgins et al., 2022). Exploring a range of data-mining and text classification methods for systematic reviews, the authors uncovered that early often employed approaches (e.g., rule-based extraction) gave way to classical machine-learning (e.g., naïve Bayes and support vector machine classifiers), and more recently, trends indicate increased application of deep learning architectures such as neural networks and word embeddings (for yearly trends in reported systems architectures, see Schmidt et al., 2021, p. 8).

In social sciences and related disciplines, several related reviews of tools and techniques for automating tasks associated with systematic reviews and meta-analyses have been conducted. Table 1 provides a summary of related research.

**Table 1.** Related literature.

Reference	Research discipline (Sample size)	Primary focus
Antons et al. (2020)	Innovation ( *n*=140)	Text mining methods in innovation research
Cairo et al. (2019)	Computer Science ( *n*=17)	ML techniques for secondary studies
Dridi et al. (2021)	Management ( *n*=124)	Scholarly data mining applications
Göpfert et al. (2022)	Multidisciplinary ( *n*=80)	Measurement extraction methods using NLP
Feng et al. (2017)	Software Engineering ( *n*=32)	Text mining techniques and tools to facilitate SLRs
Kohl et al. (2018)	Multidisciplinary ( *n*=22)	Tools for systematic reviews and mapping studies
Roldan-Baluis et al. (2022)	Multidisciplinary ( *n*=46)	NLP and ML for processing unstructured texts in digital format
Sundaram and Berleant (2023)	Multidisciplinary ( *n*=29)	Text mining-based automation of SLRs
Wagner et al. (2022)	Information Systems and related Social Sciences (NR)	Review of AI in literature reviews
Yang et al. (2023)	Education ( *n*=161)	Text mining techniques in educational research

*Note.* AI = Artificial Intelligence, ML = Machine Learning, SLR = Systematic Literature Review, NLP = Natural Language Processing, NR = Not Reported.

Based on extant reviews analyzing trends in Artificial Intelligence (AI) technologies for automating Systematic Literature Review (SLR) efforts outside of clinical domains, we noted several trends. First, techniques to facilitate abstraction, generalization, and grouping of primary studies represent the majority of (semi)automated approaches. Second, extant reviews highlight a predominant focus on supporting search and study selection stages, with significant gaps in (semi)automating data extraction. Third, evaluation concerns underscore the importance of performance metrics, validation procedures, benchmark datasets and improved transparency and reporting standards to ensure the reliability and effectiveness of AI techniques. Finally, challenges in cross-discipline transferability illuminate the need for domain-specific adaptations and infrastructures.

Existing reviews evidence the widespread application of techniques such as topic modeling, clustering, and classification to support abstraction, generalization, and grouping of primary research studies. Topic modeling, particularly Latent Dirichlet Allocation (LDA), is commonly applied to (semi)automate content analysis, facilitating the distillation of complex information into meaningful insights and identification of overarching trends and patterns across a literature corpus ( Antons et al., 2020; Dridi et al., 2021; Roldan-Baluis et al., 2022; Yang et al., 2023). Additionally, classification and clustering techniques are commonly applied for tasks such as mining article metadata and automatically grouping papers by relevance to SLR research questions are ( Feng et al., 2017; Sundaram & Berleant, 2023; Wagner et al., 2022).

(Semi)automation efforts in social sciences and related disciplines have primarily addressed supporting the search and study selection stages of SLRs ( Cairo et al., 2019; Feng et al., 2017), with significant gaps in automation techniques for tasks such as data extraction ( Göpfert et al., 2022; Sundaram & Berleant, 2023). Further, available software tools lack functionality to support activities beyond study selection ( Kohl et al., 2018). Key findings across these reviews underscore the need for more comprehensive automation solutions, particularly for quantitative data extraction ( Göpfert et al., 2022).

Additionally, researchers express transparency concerns regarding AI’s reliance on black box models ( Wagner et al., 2022) and limited visibility into underlying processes and algorithms in proprietary software solutions ( Antons et al., 2020). Adding to these considerations, Antons et al. (2020) identified substantial reporting gaps, including 35 of 140 articles omitting details about software used. Since metrics alone may not be sufficient to objectively assess AI performance ( Dridi et al., 2021), strategies for mitigating bias and ensuring transparency and fairness represent a substantial topic of automation discourse.

Ongoing research of AI tools for clinical studies ( Sundaram & Berleant, 2023) and the extraction of PICO data elements from RCTs ( Wagner et al., 2022) underscore the success of domain-specific adaptation efforts. While the promise of adopting AI-based techniques and tools in social science domains is evident ( Cairo et al., 2019; Feng et al., 2017), extant research reveals challenges in transferring existing technologies across disciplines. Further, many SLR software applications are tailored specifically for health and medical science research ( Kohl et al., 2018). Literature suggests that overcoming global obstacles can be facilitated by concentrated efforts to develop domain-specific knowledge representations, such as standardized construct taxonomies and vocabularies ( Feng et al., 2017; Göpfert et al., 2022; Wagner et al., 2022).

### Objectives

In the present study, we conduct a baseline review of existing and emergent techniques for the (semi)automated data extraction which focus on target data entities and elements relevant to evidence synthesis across social sciences research domains. We report findings that supplement the growing body of research dedicated to the automatic extraction of data from clinical and medical research.

## Methods

### Protocol registration

This LSR was conducted following a pre-registered and published protocol ( Legate & Nimon, 2023b). For additional details and project repositories, see ‘Data availability’ section.

### Living review

**Figure 1.**
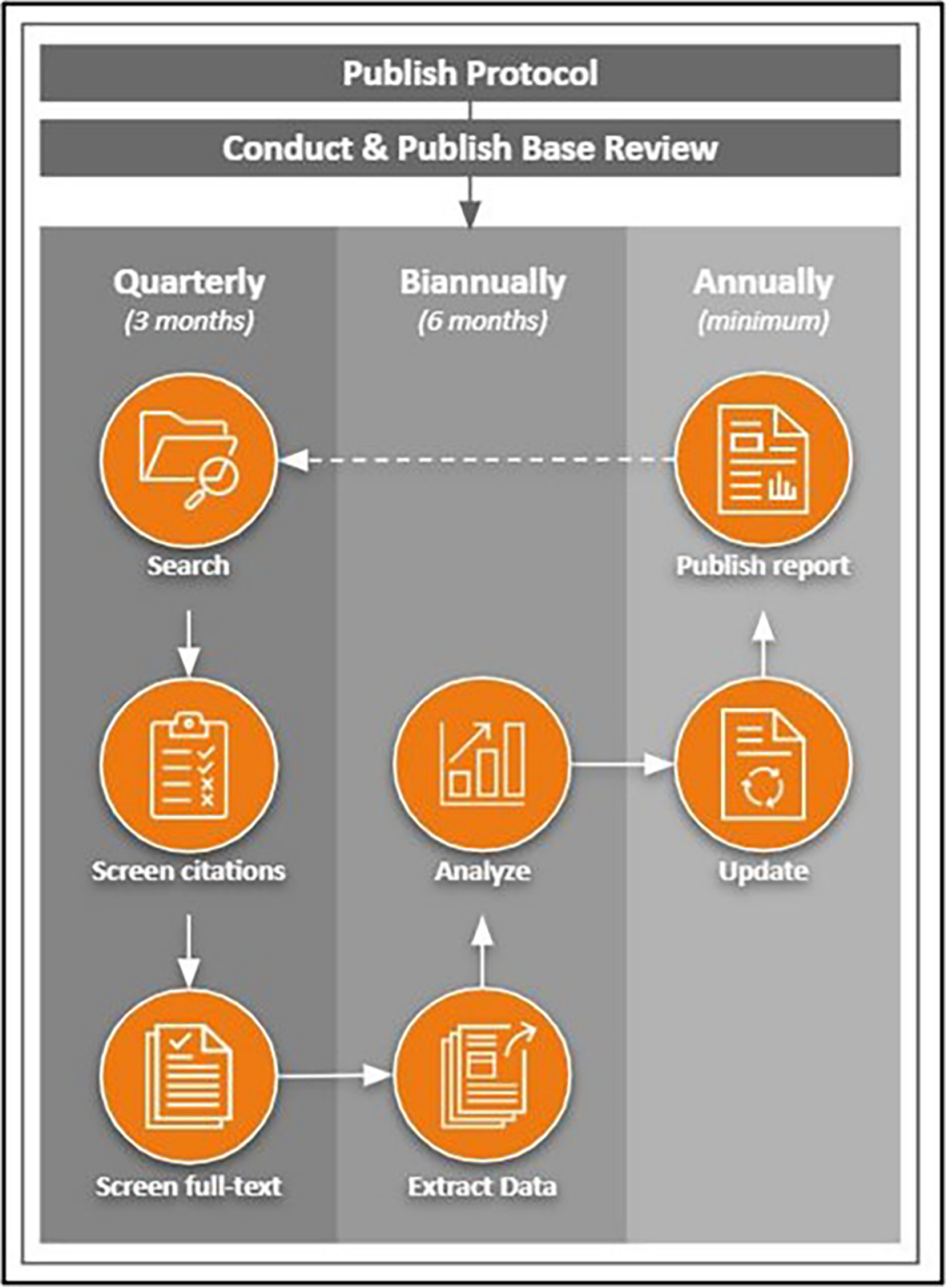
LSR workflow. This image is reproduced under the terms of a Creative Commons Attribution 4.0 International license (CC-BY 4.0) from Legate and Nimon (2023b). *Note.* Arrows represent stages involved in a static systematic review; the dotted line (from “Publish Report” to “Search”) represents the stage at which the review process is repeated from the beginning while the review remains in living status.

We adopted the LSR methodology for this study primarily due to the pace of emerging evidence, particularly in light of ongoing technological advancements. The ongoing nature of an LSR allows for continuous surveillance, ensuring timely presentation of new information that may influence findings ( Elliott et al., 2014, 2017; Khamis et al., 2019). This baseline review was initiated upon peer approval of the associated protocol ( Legate & Nimon, 2023b). It remains our intent for the review to be continually updated via living methodological surveys of published research ( Khamis et al., 2019) following the workflow schedule as previously published in the protocol (see Figure 1; Legate & Nimon, 2023b). Necessary adjustments to the workflow will be detailed within each subsequent update.

### Eligibility criteria

As in prior reviews, English language reports, published 2005 or later were considered for inclusion ( Jonnalagadda et al., 2015; O’Mara-Eves et al., 2015; Schmidt et al., 2020). Eligible studies utilized, presented, and/or evaluated semi-automated approaches to support evidence-synthesis research methods (e.g., systematic reviews, psychometric meta-analyses, meta-analyses of effect sizes, etc.). Studies may have reported on any automated approach to data extraction, given that at least one entity was extracted semi-automatically from the abstracts or full-text of a literature corpus and sufficient detail was reported for:

a) entity(ies) or data element(s) extracted;
b) location of the extracted entities (e.g., abstract, methods, results sections); and
c) the automation tool and/or technique used to support extraction.

We excluded studies labeled as editorials, briefs, or opinion pieces and/or engaged in narrative discussion without applying automation tools or technologies to extract data from research literature. Per the protocol, we also excluded studies that applied tools/techniques to:

a) extract data exclusively from medical, biomedical, clinical (e.g., RCTs), or natural science research;
b) extract metadata only (i.e., citation details) from research articles; or
c) extract data from alternative (i.e., non-research literature) sources (e.g., web scraping, electronic communications, transcripts, etc.).

### Search sources

The search strategy for this review was developed by adapting the search strategy from a related LSR of clinical research ( Schmidt et al., 2020). We initially intended to conduct searches in the same databases used by Schmidt et al. (2020, 2021), excluding medical research sources. Because *IEEE* content is indexed in *Web of Science* ( Young, 2023), we did not include *IEEE Xplore* as a separate source. We added two additional databases ( *ACL* and *ArXiv*) and conducted a search for data extraction tools in the Systematic Review Toolbox ( Marshall et al., 2022) to capture associated articles. Our final source list of sources for this baseline review included: (1) *ACL,* (2) *ArXiv*, (3) *DBLP Computer Science Bibliography*, (4) *Web of Science Core Collection*, and (5) *SR Toolbox.* The Web of Science search and deduplication followed procedures stated in the protocol ( Legate & Nimon, 2023b). We adapted source code developed by Schmidt et al. (2021) for automating search, retrieval, and deduplication functions on full database dumps for *ACL*, *ArXiv*, and *DBLP* platforms. Search syntax and adapted source code are available in the project repository (see ‘Data availability’ section).

### Study selection

Title, abstract, and full-text screening was conducted using Rayyan ( Ouzzani et al., 2016; free and subscription accounts available at https://www.rayyan.ai/). Three researchers (1000 abstracts per week) screened all titles and abstracts. Researchers met weekly to review, resolve conflicts, and further develop the codebook for this LSR. All conflicts that arose during the title and abstract screening ( *n*=103/ *N*=10,644) were resolved on a weekly basis. Where disagreements arose, they were related to methods for abstractive text summarization and transferability of methods applied to clinical research studies (i.e., RCTs). In cases where level of abstraction and potential for transferability could not be determined from the abstract alone, full text articles were reviewed and discussed by all three researchers until consensus was reached.

For the data extraction stage, a Google form was developed following items of interest as described in the protocol. All data extraction tasks were performed independently in triplicate. Researchers met weekly to review and reach a consensus on coding of extracted items of interest. The extraction form was updated over the course of data extraction to better fit project goals and promote reliability of future updates.

We originally intended to conduct Inter-Rater Reliability (IRR) assessments to provide reliability estimates following each stage of the baseline review ( Belur et al., 2018; Zhao et al., 2022). Given the nascency of our research and scope of our items of interest, coding forms allowed for input of “other” responses (e.g., APA data elements) that were not included in extant reviews that focus on medical and clinical data extraction (e.g., PICO elements). Further, data extraction presented opportunities to develop reporting structure for methods and items of interest that were not reported in prior literature (e.g., NER, open-source tools). A weekly review meeting was used to continually develop the project codebook to promote continuity, structure, and develop an IRR framework for future iterations of this review.

## Results

### Search results

**Figure 2.**
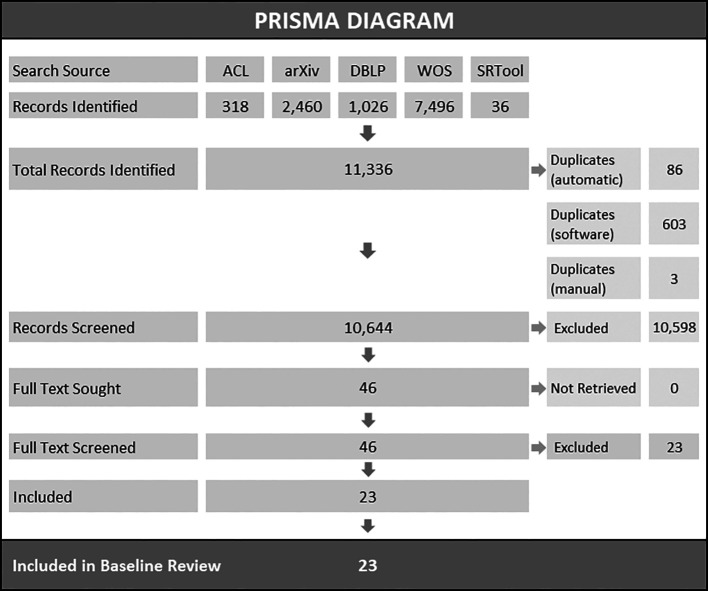
PRISMA diagram. *Note.* ACL=ACL Anthology ( https://aclanthology.org/), arXiv=arXiv Research-Sharing Platform ( https://arxiv.org/), DBLP=DBLP Computer Science Bibliography ( https://dblp.org/), WOS=Web of Science Core Collection, SRTool=Systematic Review Toolbox ( http://systematicreviewtools.com/).

Search results are presented in the PRISMA flowchart (see Figure 2). A total of 11,336 records were identified through all search sources, including databases and publications available through the SRToolbox ( Marshall et al., 2022). After deduplication, 10,644 articles were included in the title and abstract screening stage. We retrieved 46 articles for full-text screening. One duplicate print was detected during full text screening and was removed. This iteration of the LSR includes 23 articles. Detailed description of deduplication and preliminary screening procedures are available in the OSF project repository (see ‘Data availability’ section).

**Figure 3.**
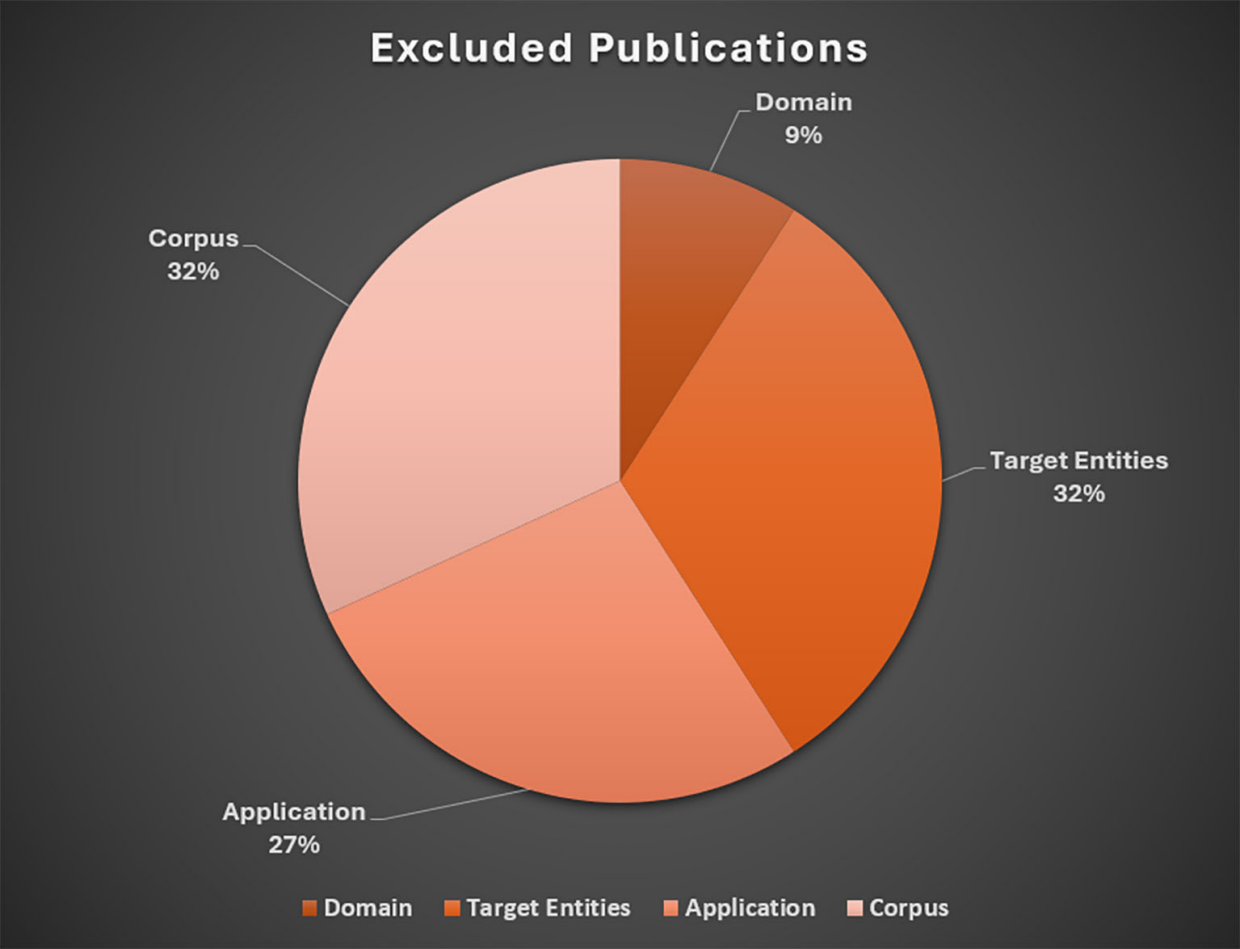
Excluded publications. *Note.* Domain = exclusively medical, biomedical, clinical, or natural science ( *n*=2); Target entities = Lack of detail in reporting extracted entities ( *n*=7); Application = no application, testing, or extraction conducted manually ( *n*=6); Lack of Detail in Reporting Corpus or Wrong Corpus ( *n*=7).

**Figure 4.**
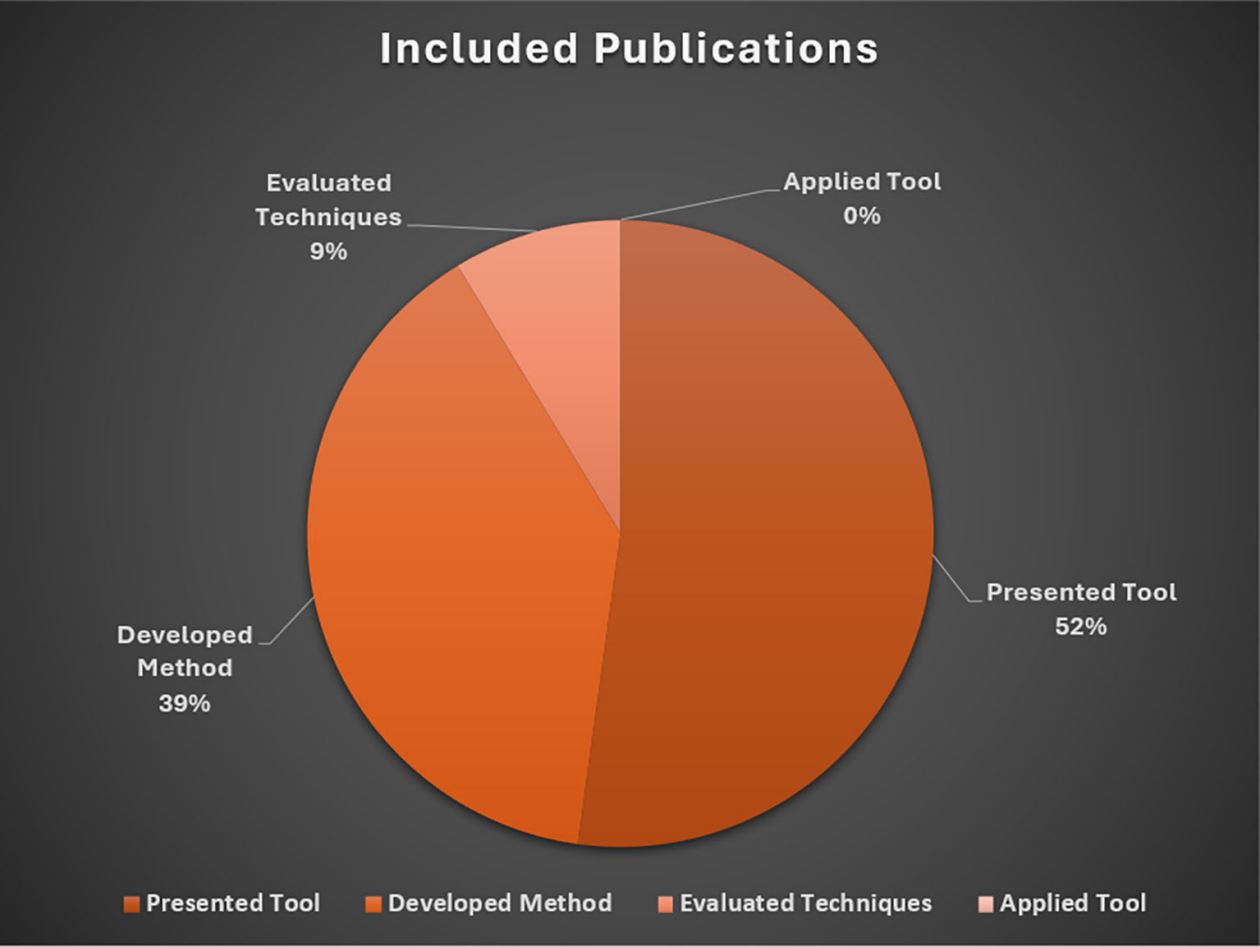
Included publications. *Note.* Presented Tool = Describe/demonstrated a software tool, system, or application for data extraction ( *n*=12), Developed Method = Developed techniques and/or methods for automated data extraction ( *n*=9); Evaluated Techniques = Tested or evaluated the performance of existing tools, techniques, or methods ( *n*=2); Applied Tool = Applied automation tools to conduct secondary research ( *n*=0).

The following sections describe the rationale for exclusions, followed by a brief overview of studies included in the baseline review. These results are presented in Figures 3 and 4, respectively. An overview of included studies is presented in Table 2.

**Table 2.** Included studies.

Title	Reference	Summary description
A model for the identification of the functional structures of unstructured abstracts in the social sciences	Shen et al. (2022)	Proposed a high-performance model for identifying functional structures of unstructured abstracts in the social sciences.
A Semi-automatic Data Extraction System for Heterogeneous Data Sources: a Case Study from Cotton Industry	Nayak et al. (2021)	Proposed a novel data extraction system based on text mining approaches to discover relevant and focused information from diverse unstructured data sources.
An interactive query-based approach for summarizing scientific documents	Bayatmakou et al. (2022)	Proposed an interactive multi-document text summarization approach that allows users to specify composition of a summary and refine initial query by user-selected keywords and sentences extracted from retrieved documents.
Automatic results identification in software engineering papers. Is it possible?	Torres et al. (2012)	Analyzed existing methods for sentence classification in scientific papers and evaluates their feasibility in unstructured papers in the Software Engineering area.
Contextual information retrieval in research articles: Semantic publishing tools for the research community	Angrosh et al. (2014)	Introduced conceptual framework (and linked data application) for modeling contexts associated with sentences and converting information extracted from research articles into machine-understandable data.
CORWA: A Citation-Oriented Related Work Annotation Dataset	Li et al. (2022)	Presented new approach to related work generation in academic research papers and introduced annotation dataset for labeling different types of citation text fragments from various information sources.
DASyR (IR) - Document Analysis System for Systematic Reviews (in Information Retrieval)	Piroi et al. (2015)	Introduced a semi-automatic document analysis system/framework for annotating published papers for ontology population, particularly in domains lacking adequate dictionaries.
Detecting In-line Mathematical Expressions in Scientific Documents	Iwatsuki et al. (2017)	Reported preliminary results applying a method for identifying in-line mathematical expressions in PDF documents utilizing both layout and linguistic features.
Extracting the characteristics of life cycle assessments via data mining	Diaz-Elsayed and Zhang (2020)	Proposed a method for automatically extracting key characteristics of life cycle assessments (LCAs) from journal articles.
Machine Reading of Hypotheses for Organizational Research Reviews and Pre-trained Models via R Shiny App for Non-Programmers	Chen et al. (2021)	Introduced NLP models for accelerating the discovery, extraction, and organization of theoretical developments from social science publications.
MetaSeer.STEM: Towards Automating Meta-Analyses	Neppalli et al. (2016)	Proposed a machine learning-based system developed to support automated extraction of data pertinent to STEM education meta-analyses.
Mining Social Science Publications for Survey Variables	Zielinski and Mutschke (2017)	Described a work-in-progress development of new techniques or methods for identifying variables used in social science research.
Ontology-based and User-focused Automatic Text Summarization (OATS): Using COVID-19 Risk Factors as an Example	Chen et al. (2020)	Proposed an ontology-based system which users could access and utilize for automatically generating text summarization from unstructured text.
Ontology-Driven Information Extraction from Research Publications	Pertsas and Constantopoulos (2018)	Introduced a system designed to extract information from research articles, associate it with other sources, and infer new knowledge.
Research Method Classification with Deep Transfer Learning for Semi-Automatic Meta-Analysis of Information Systems Papers	Anisienia et al. (2021)	Presented an artifact that uses deep transfer learning for multi-label classification of research methods for an Information Systems corpus.
Scaling Systematic Literature Reviews with Machine Learning Pipelines	Goldfarb-Tarrant et al. (2020)	Described a pipeline that automates three stages of a systematic review: searching for documents, selecting relevant documents, and extracting data.
Searching for tables in digital documents	Liu et al. (2007)	Introduced an automatic table extraction and search engine system.
Section-wise indexing and retrieval of research articles	Shahid and Afzal (2018)	Described development and evaluation of a technique for tagging paper's content with logical sections appearing in scientific documents.
Sysrev: A FAIR Platform for Data Curation and Systematic Evidence Review	Bozada et al. (2021)	Introduced a platform for aiding in systematic reviews and data extraction by providing access to digital documents and facilitating collaboration in research projects.
Team EP at TAC 2018: Automating data extraction in systematic reviews of environmental agents	Nowak and Kunstman (2019)	Presented a solution for automating data extraction in systematic reviews of environmental agents.
The Canonical Model of Structure for Data Extraction in Systematic Reviews of Scientific Research Articles	Aliyu et al. (2018)	Developed a canonical model of structure approach that identifies sections from documents and extracts the headings and subheadings from the sections.
Towards a Semi-Automated Approach for Systematic Literature Reviews Completed Research	Denzler et al. (2021)	Presented a flexible and modifiable artifact to support systematic literature review processes holistically.
UniHD@CL-SciSumm 2020: Citation Extraction as Search	Aumiller et al. (2020)	Presented method to identify references from citation text spans and classify citation spans by discourse facets.

### Excluded publications

Most studies were excluded due to lack of detail in extracted data entities ( *n*=7) and wrong corpus or data source ( *n*=7). Carrión-Toro et al. (2022), for example, developed a method and software tool supporting researchers with selection of relevant key criteria in a field of study based on term frequencies. While text summarization has proven valuable for evidence synthesis tasks, the primary focus of this LSR involves efforts to extract specific data points from primary research ( O’Connor et al., 2019). We also excluded extraction techniques that were not applied to abstracts or full text of research articles. Ochoa-Hernández et al. (2018), for instance, presented a method to automatically extract concepts from web blog articles.

The second most common exclusion category were articles that presented techniques or systems utilizing pre-extracted data ( *n*=4). Ali and Gravino (2018), for example, proposed an ontology-based SLR system with semantic web technologies; however, the data (derived from a prior review conducted by the authors) were added to the ontology system after the manual extraction stage. Finally, articles were excluded due to exclusive application in medical/clinical research ( *n*=2), or the proposed tool had not yet been implemented ( *n*=2). Goswami et al. (2019), for example, described and evaluated a supervised ML framework to identify and extract anxiety outcome measures from clinical trial articles. Zhitomirsky-Geffet et al. (2020) presented a conceptual description of a network-based data model capable of mining quantitative results from social sciences articles, but the system had not been implemented at the time of publication.

### Included publications

The majority of included studies ( *n*=12) presented or described a software tool, system, or application to support researchers extracting data from research literature. The second most common inclusion category focused on the development of specialized techniques or methods for automating data extraction tasks ( *n*=9). We identified two studies that evaluated or tested the performance of existing tools or methods for (semi)automated data extraction. Unlike related reviews of data extraction methods for healthcare interviews (see Schmidt et al., 2023), we did not identify social science studies applying existing automated data extraction tools to conduct secondary research.

### Automated approaches

To report approaches identified, we organized the extracted data under four overarching categories, including: (1) data preprocessing and feature engineering, (2) model architectures and components, (3) rule-bases, and (4) evaluation metrics. See ‘Data Availability’ section for labeling and additional descriptions of techniques. We opted to extract and report rule-based techniques separately because the approaches we identified intertwined with various aspects of the data processing and extraction pipeline, spanning data preprocessing to the model architecture itself. This distinction allows for more discussion about the prevalence, scope and utility of these techniques.

### Data preprocessing and feature engineering

**Figure 5.**
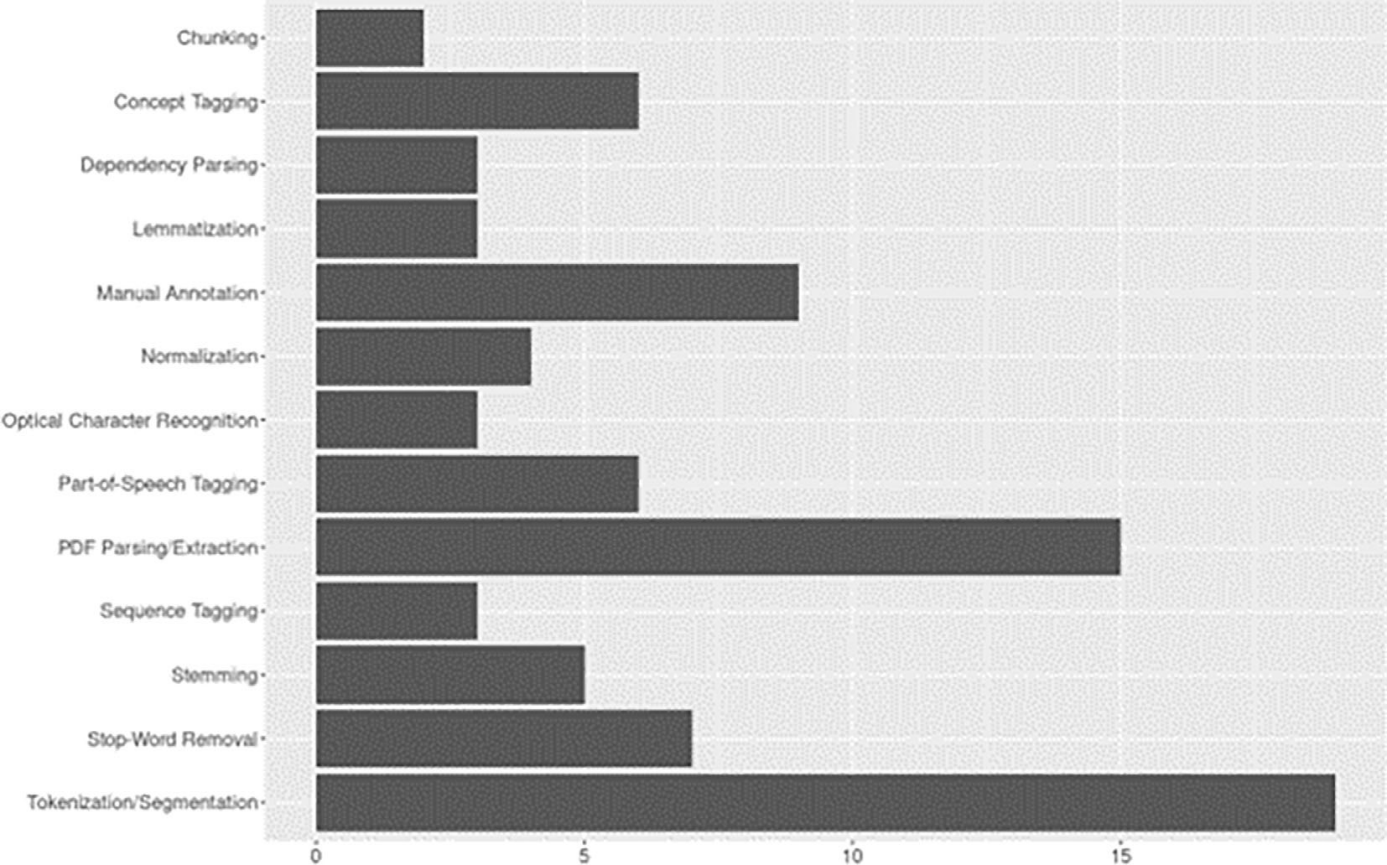
Data preprocessing.

The data preprocessing category encompasses methods and techniques used to preprocess raw text and data before it is fed into ML/NLP models. This includes tasks such as tokenization, stemming, lemmatization, stop word removal, and other steps necessary to clean and prepare the text data for analysis. Figure 5 plots the aggregate results of preprocessing techniques identified.

Nearly all studies applied tokenization and/or segmentation (83%, *n*=19) for breaking down text into manageable units. Similarly, PDF parsing/extraction techniques were applied in 65% ( *n*=15) of studies, the remaining studies applied extraction to other document formats (e.g., journal articles available online in HTML format; see Diaz-Elsayed & Zhang, 2020). While similar methods, which additionally take into account syntactic structure, including chunking and dependency parsing were less frequently applied ( Angrosh et al., 2014; Li et al., 2022; Nayak et al., 2021; Pertsas & Constantopoulos, 2018). Tagging methods, including PoS tagging (assigning grammatical categories, e.g., noun, verb), followed by concept tagging (e.g., semantic annotation), or sequence tagging, where labels were assigned based on order of appearance, were used in 43% ( *n*=15) of studies. Nine studies used manual annotation for training and/or evaluation.

Among noise reduction approaches, stop-word removal was the most common, stemming, normalization, and lemmatization were applied, though less frequently. For stemming approaches, the Porter stemmer ( Porter, 1980), including its extensions (e.g., Porter2, S-stemmer, snowball stemmer), were as commonly reported as traditional stemmers (see Aumiller et al., 2020; Bayatmakou et al., 2022; Shahid & Afzal, 2018). Optical Character Recognition (OCR) appeared in three studies, however, Iwatsuki et al. (2017) used OCR only as a benchmark for evaluating their CRF method for detecting math expressions.

**Figure 6.**
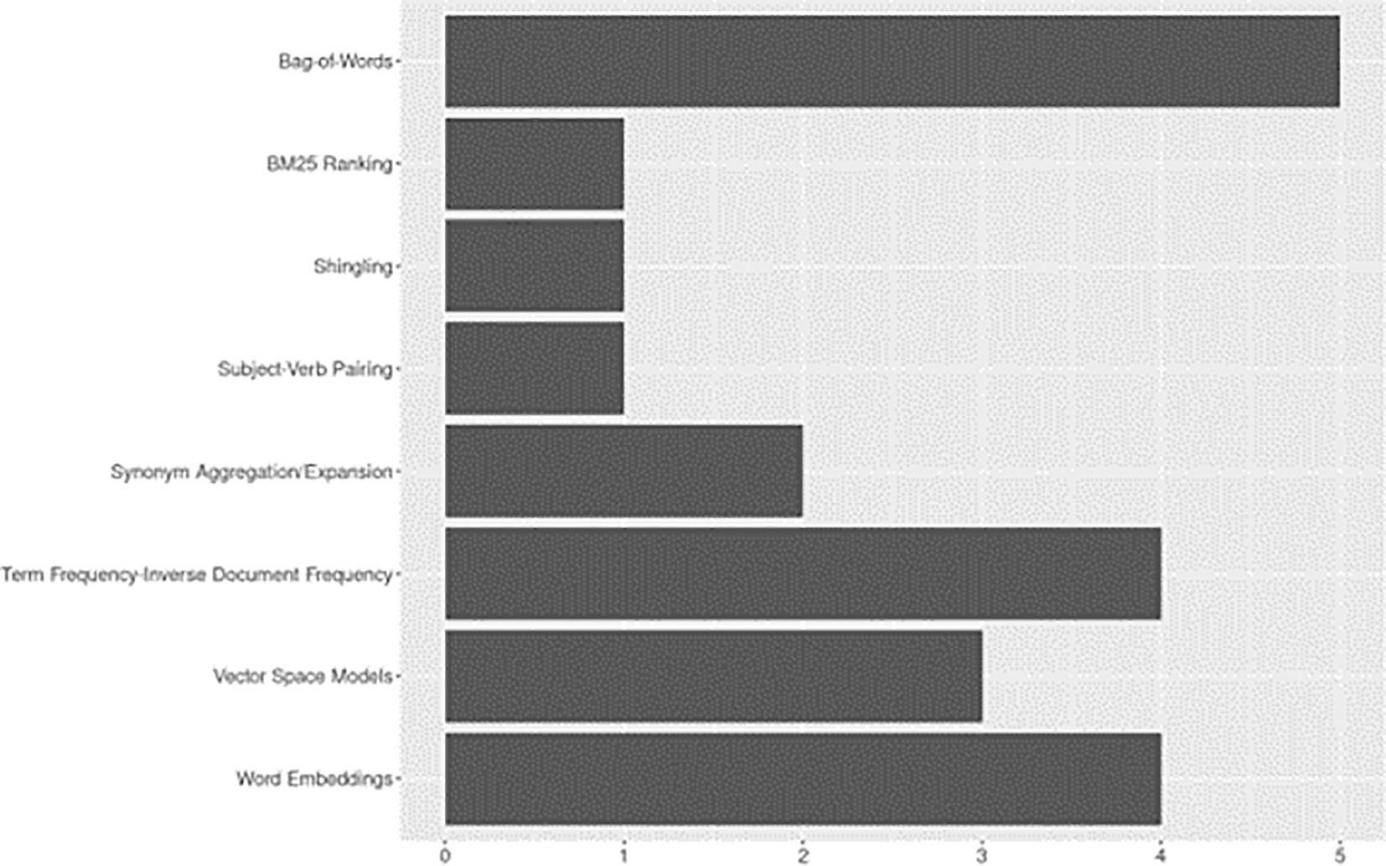
Feature engineering.

Feature engineering (e.g., ranking functions, representation learning and feature extraction techniques) covers a range of methods essential for transforming raw text data into structured, machine-readable representations to facilitate downstream ML/NLP tasks ( Kowsari et al., 2019). See Figure 6.

Word embeddings were the most frequently used techniques. We grouped ELMo (word embeddings from language models) with traditional word embeddings such as Word2Vec and Glove ( Kowsari et al., 2019; Young et al., 2018, Chapter 6). Of these, GloVe was used in four studies ( Chen et al., 2021; Goldfarb-Tarrant et al., 2020; Nowak & Kunstman, 2019; Anisienia et al., 2021) and ELMo in two ( Nowak & Kunstman, 2019; Anisienia et al., 2021). The most common frequency-based feature representation approaches were Bag-of-Words (BoW, *n*=5) and Term frequency-Inverse Document Frequency (TF-IDF, *n*=4). Although less frequently applied in the corpus, methods for representing words or documents as vectors based on semantic properties such as Vector Space Models (VSM) and sentence embeddings were used as early as 2007. Other less commonly reported methods included synonym aggregation/expansion, best match ranking (BM25), shingling, and subject-verb pairings.

### Model architectures and components

**Figure 7.**
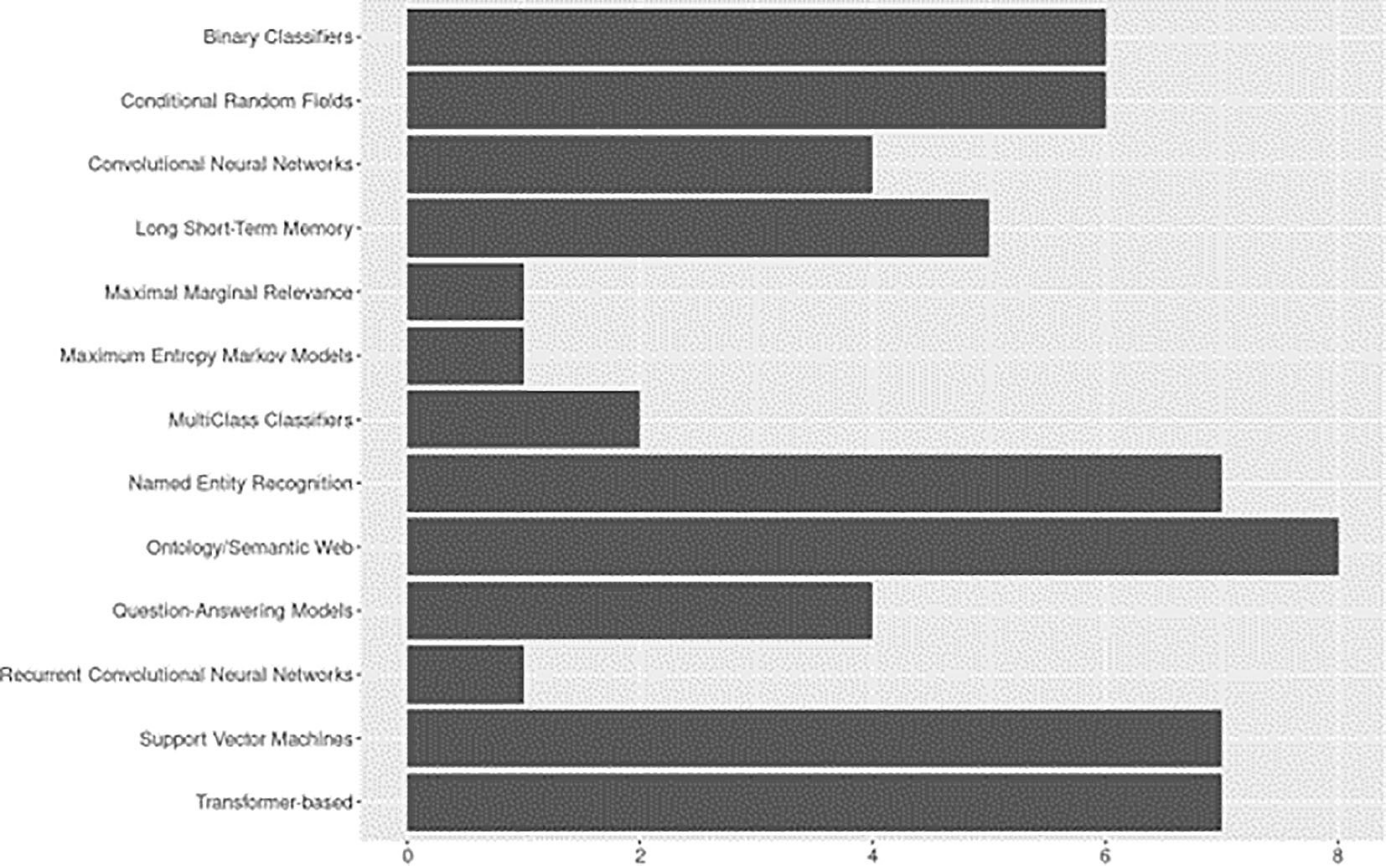
Model architectures and components.

The model architecture category focuses on the architectures and components of ML/NLP models used for data extraction. Results are shown in Figure 7. Some approaches overlapped across applications – e.g., semantic web or semantic indexing structures and ontology-pipeline approaches – we grouped these techniques into categories to facilitate reporting. Likewise, all transformer-based approaches were grouped into a single category, however, specific architectures and components are discussed in the sections below, and detailed coding of extracted data is available in the supplemental data files (see ‘Underlying Data’ section). Where ruled-based approaches were considered a part of the system architecture, they are reported under the ‘Rule-bases’ section.

Overall, approaches ranged from straightforward implementations to complex layered architectures. Examples of more straightforward approaches included architectures based entirely on rule-bases (e.g., Diaz-Elsayed & Zhang, 2020), applications based one classification method (e.g., naïve Bayes; Neppalli et al., 2016), or those utilizing a single type of probabilistic model ( Angrosh et al., 2014; Iwatsuki et al., 2017). At the other end of the complexity continuum, Nowak and Kunstman (2019) presented an end-to-end deep learning model based on a BI-LSTM-CRF architecture with interleaved alternating LSTM layers and highway connections. In the following sections, we further elaborate on various approaches identified.

***Ontology-based and Semantic Web.*** These pipelines involve leveraging ontologies and semantic web technologies for semantic annotation or knowledge representation. Among included studies, ontology and semantic web capabilities were explored as early as 2014, but the preliminary results from this baseline review suggest an upward trend in recent years. Angrosh et al. (2014), for example, developed a Sentence Context Ontology (SENTCON) for modeling the contexts of information extracted from research documents. Piroi et al. (2015) developed and presented an annotation system for populating ontologies in domains lacking adequate dictionaries. Some work focused on automatically mapping structures of research documents. For example, using an open source lexical database to develop a canonical model of structure, Aliyu et al. (2018) were able to automatically identify and extract target paper sections from research documents. Shahid and Afzal (2018) utilized specialized ontologies to automatically tag content in research papers by logical sections. Chen et al. (2020) presented a novel framework for text summarization, including ontology-based topic identification and user-focused summarization modules.

***Transformer-based Approaches.*** Our results suggested that transformer-based approaches have experienced rapid growth since 2020. Bidirectional Encoder Representations from Transformers (BERT) and other BERT-based language models made up the majority of transformer-based approaches. Specifically BERT ( Aumiller et al., 2020; Shen et al., 2022) and SciBERT ( Goldfarb-Tarrant et al., 2020; Li et al., 2022) were the most utilized for tasks relevant to extracting data from research in social sciences. Others language models included BioBERT ( Chen et al., 2020) and distilBERT ( Goldfarb-Tarrant et al., 2020). We identified a recent application of the Hugging Face LED model ( Li et al., 2022), a pre-trained longformer model developed to address length limitations associated with other transformer-based approaches (see Beltagy et al., 2020).

***Named Entity Recognition (NER).*** Six of the included studies applied Named Entity Recognition (NER) techniques. Increasing availability of tools to support the entire SLR pipeline, including data extraction efforts, may be partially to credit for upward trends in NER applications. Based on applications we identified, NER would best be described as versatile. Some studies incorporated NER as an integral component embedded throughout a larger ML/NLP pipeline (e.g., Goldfarb-Tarrant et al., 2020), others included NER subcomponents leveraged primarily for preprocessing and feature representation tasks (e.g., Pertsas & Constantopoulos, 2018), and in one study, authors took advantage of open source NER tools that could be easily integrated into a highly modifiable artifact serving as platform for future development of holistic approaches to scaling SLR tasks (e.g., Denzler et al., 2021).

***Extractive Questing-Answering Models.*** Extractive questing-answering models involve tasks where a model generates answers to questions based on a given context. Question-answering models appeared in our dataset as early as 2007 ( Liu et al., 2007), with the remaining applications published in 2020 or later. Question answering techniques have a range of applications that most readers are likely familiar with, like chatbots and intelligent assistants (e.g., Alexa, Google Assistant, Siri). However, state-of-the-art approaches for question-answering over knowledge bases are also being put to use in the data extraction arena. The study by Bayatmakou et al. (2022), for example, introduced new methods for interactive multi-document text summarization that allow users to specify summary compositions and interactively refine queries after reviewing complete sentences automatically extracted from documents.

***Classifiers.*** For classification approaches, we followed Schmidt et al. (2021) in reporting instances of Support Vector Machines (SVM) separately from other binary classifiers and likewise found a high prevalence of SVM usage, accounting for 50% of all binary classifiers identified ( Goldfarb-Tarrant et al., 2020; Shahid & Afzal, 2018; Shen et al., 2022; Zielinski & Mutschke, 2017). Among classifiers that use a linear combination of inputs ( Jurafsky & Martin, 2024), naïve Bayes was the most frequent ( Neppalli et al., 2016; Shahid & Afzal, 2018; Torres et al., 2012; Zielinski & Mutschke, 2017). One study used a Perceptron classifier; however, it was extended (i.e., OvR) to handle multiclass problems ( Aumiller et al., 2020). Multi-class classifiers were less common with one instance each of k-Nearest Neighbors (aka KNN/kLog; Zielinski & Mutschke, 2017) and the J48 classifier (C4.5 Decision Trees; Piroi et al., 2015).

***Neural Networks.*** Overall, there were a variety of neural network applications across the included studies. Most used Long Short-term Memory (LSTM), more specifically, Bidirectional LSTM (BiLSTM). We also identified one application Bidirectional Gated Recurrent Unit (BiGRU; Shen et al., 2022). Convolutional Neural Network (CNN) architectures ( Goldfarb-Tarrant et al., 2020; Nowak & Kunstman, 2019; Anisienia et al., 2021) were also present. Several studies evaluated state-of-the-art deep learning methods. For example, Shen et al. (2022) compared the performance of deep learning models (TextCNN and BERT) for sentence classification in social sciences abstracts. In another comparative study, Anisienia et al. (2021) compared methods for pretraining deep contextualized word representations for cutting-edge transfer learning techniques based on CNN and LSTM architectures in addition to classifier models (e.g., SVM).

***Probabilistic Models.*** Among probabilistic models, Conditional Random Field (CRF) applications were predominant in our dataset. CRF was often applied for sequence labeling tasks, such as named entity recognition (e.g., Nayak et al., 2021), or for classification tasks (e.g., Angrosh et al. 2014). Overall, included studies provided evidence that CRF can form a powerful architecture when combined with RNNs (e.g., bi-GRU-CRF, bi-LSTM-CRF; see Nowak & Kunstman, 2019; Shen et al., 2022). We found a single application of the Maximum Entropy Markov Model (MEMM), however, based on experimental results the authors ultimately selected CRF for identifying sentence context for extraction from research publications ( Angrosh et al., 2014).

### Rule-bases

**Figure 8.**
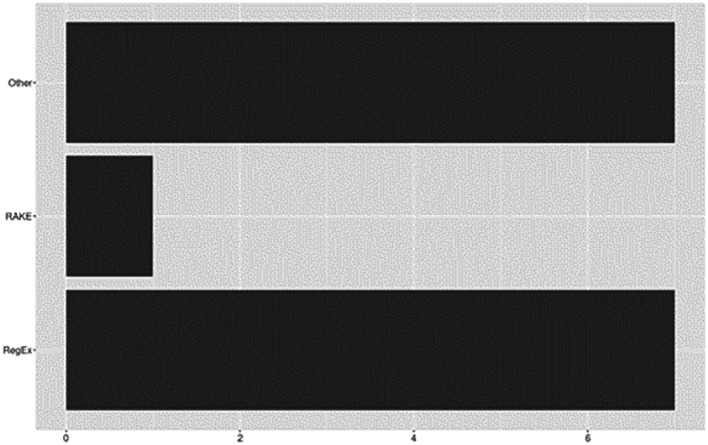
Rule-bases.

Rule-based techniques involve the application of predefined rules or patterns to extract specific features from the text. Versatile and widely applicable, they offer a robust framework for automating data extraction or for capturing relevant information from large volumes of text. See Figure 8 for rule-based approaches reported across included studies.

Overall, 70% ( *n*=16) of included studies utilized rule- or heuristic-based approaches to support a variety of tasks for data extraction. Of these, nearly half ( *n*=7) reported using Regular Expressions (RegEx). For example, based on rules developed from manual inspection, RegEx was used by Torres et al. (2012) to construct patterns for identifying specific types of sentences (e.g., objective, results, conclusions) and by Goldfarb-Tarrant et al. (2020) for splitting papers into specific sections (e.g., abstract, introduction, methods). Alternatively, Pertsas and Constantopoulos (2018) used RegEx to exploit lexico-syntactic patterns derived from an ontology knowledge base (Activities, Goals, and Propositions). Other RegEx uses included modifying datasets to incorporate patterns related to citation mentions ( Anisienia et al., 2021) or application of rule-based chunking and processing to identify and extract relevant chunks from text ( Nayak et al., 2021). The remaining six studies described custom rule-based algorithms or other heuristic approaches. Li et al. (2022), for example, applied rule-based algorithms PrefixSpan and Gap-Bide for the extraction of frequent discourse sequences. RAKE (Rapid Automatic Keyword Extraction) was applied by Bayatmakou et al. (2022) to extract keywords which served as representations of a document’s content. And Aliyu et al. (2018) described a rule-based algorithm developed for processing full-text documents to identify and extract section headings.

### Evaluation metrics

**Figure 9.**
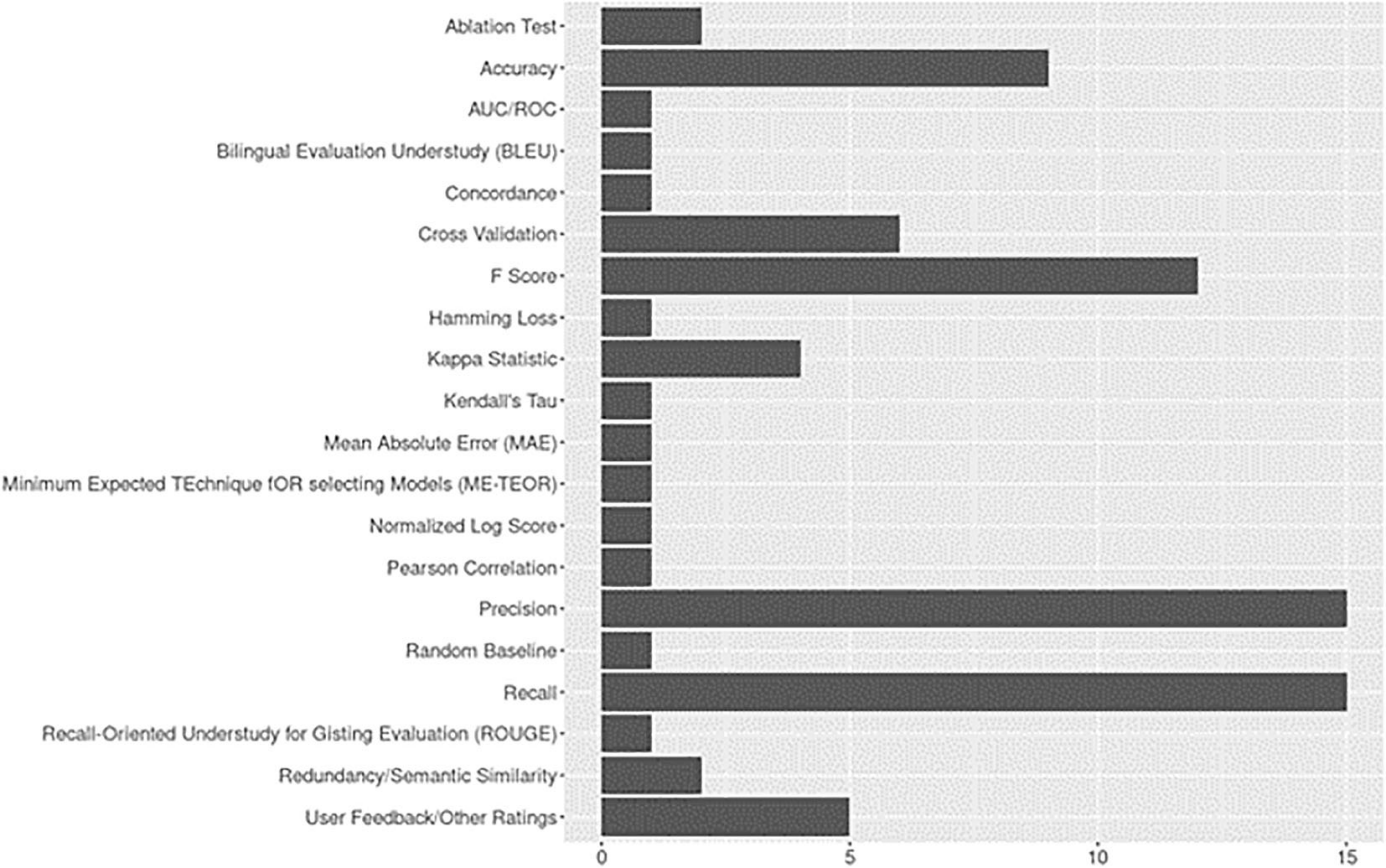
Evaluation metrics.

Evaluation metrics are presented in Figure 9. Precision, recall, F-scores, and accuracy were predominantly reported across studies, including the earliest published articles. For assessment of model performance, six studies used cross-validation (CV), a process of “averaging several hold-out estimators of the risk corresponding to different data splits” ( Arlot & Celisse, 2010, p. 53). *K*-fold CV (5 or 10 folds) was predominantly applied ( Angrosh et al., 2014; Iwatsuki et al., 2017; Neppalli et al., 2016; Shen et al., 2022, with one application of leave-one-out or LOOCV ( Piroi et al.; 2015) and one application of document level CV used as a supplemental technique to *k*-fold ( Neppalli et al., 2016). Five studies provided description of user feedback and other ratings. User feedback (among other metrics) was reported by Li et al. (2022) who conducted expert human comparative assessment to assess fluency, relevance, coherence, and overall quality of model citation span/sentence generation outputs. This category also included evaluation metrics not listed in the sources we adapted when developing our protocol (see O’Mara-Eaves et al., 2015, p. 3, Table 1; Schmidt et al., 2021, pp. 8-9). For example, in assessing their system on values returned for queries of interest, Nayak et al. (2021) reported suitably, adaptability, relevance scores, and data-dependencies. As another example, Denzler et al. (2021, p. 5) evaluated their artifact based on design science aspects (i.e., validity, efficacy, and utility).

Given the rapid growth of domain-specific ontologies and pre-trained language models, it is not surprising to find Kappa statistics reported for tasks such as evaluating agreement between human annotators when creating gold standard datasets for training and evaluation (Cohen’s Kappa, see Pertsas & Constantopoulos, 2018; Mezzich’s Kappa or Gwet’s AC1, see Anisienia et al., 2021). Semantic similarity scores, which can be used to compare model generated responses against ground truth responses in query-based or question-answering based applications, were reported in two studies (Jaccard Index, Bayatmakou et al., 2022; DKPro Similarity, Zielinski & Mutschke, 2017).

### Availability, accessibility and transferability

While only one study we reviewed presented an existing tool that was accessible to users through an online application ( sysrev.com; Bozada et al., 2021) at the time of conducting this baseline review, two other studies were either being prepared or were available through other means. These included the Holistic Modifiable Literature Review tool ( Denzler et al., 2021), which was listed as, “currently being prepared” (available at https://holimolirev.github.io/HoliMoLiRev/) and HypothesisReader ( Chen et al., 2021), which was available to users through an Rshiny application. SysRev ( Bozada et al., 2021) was also the only tool cataloged in the SR Toolbox ( Marshall et al., 2022). Six of the twenty-three studies (26%) made source code openly available ( Chen et al., 2021; Denzler et al., 2021; Diaz-Elsayed & Zhang, 2020; Goldfarb-Tarrant et al., 2020; Iwatsuki et al., 2017; Li et al. 2022). Article references and corresponding repositories are detailed in Table 3. GitHub stood out as the most popular repository for code and data sharing, and one study made source code available online through an open access publisher.

**Table 3.** Code repositories.

Reference	Code repository
Chen et al. (2021)	devtools::install_github(“canfielder/HypothesisReader”)
Denzler et al. (2021)	GitHub Repository: https://github.com/HoliMoLiRev/HoliMoLiRev
Diaz-Elsayed and Zhang (2020)	https://methods-x.com/article/S2215-0161(20)30224-7/fulltext#supplementaryMaterial
Goldfarb-Tarrant et al. (2020)	https://github.com/seraphinatarrant/systematic_reviews
Iwatsuki et al. (2017)	https://github.com/Alab-NII/inlinemath
Li et al. (2022)	https://github.com/jacklxc/CORWA

### Transferability

In the evolving landscape of systematic reviews and meta-analyses, the adaptability of tools and technologies to new research domains emerged as a critical factor for enhancing research efficiency and scope. The insights provided by many of the authors working towards automation of data extraction illuminate the transferability of various tools and technologies for research targeting the extraction of data elements beyond PICO.

Several authors of reviewed studies specifically addressed transferability in describing the development of their tools, and further subjected these tools to rigorous testing aimed at validating transferable capabilities ( Chen et al., 2020; Goldfarb-Tarrant et al., 2020; Neppalli et al., 2016). For instance, Neppalli et al. (2016) created MetaSeer.STEM with a focus on extraction of data across a range of research domains, including education, management, and health informatics. Chen et al. (2020) highlighted the adaptability of OATS, showcasing its broader application potential to fields beyond the authors’ COVID-19 specific demonstration. Finally, Goldfarb-Tarrant et al. (2020) affirmed the domain-independent nature of their framework, suggesting its suitability for various systematic reviews.

Additionally, other studies highlighted the need for transferability and discussed the potential for their research tools and technologies to be extended and adapted across varying domains, stressing the importance of flexible design principles in the development of these tools ( Angrosh et al., 2014; Diaz-Elsayed & Zhang, 2020). Angrosh et al. (2014) explained how SENTCON’s preliminary design was applied to a specific set of articles in computer science but emphasized that the tool was flexible enough to be applied to other domains through the use of the Web Ontology Language (OWL). Diaz-Elsayed & Zhang (2020) presented methods that were initially applied to wastewater-based resource recovery, but likewise emphasized that the tool was capable of evaluating other engineered systems and retrieving different types of data than those initially extracted.

As noted by Chen et al. (2021), while efforts are being made to assist the process of conducting systematic reviews there is often limited generalizability of domain-specific pre-trained language models. Many studies included in our review dedicated discussion points toward addressing the critical issue of generalizability and transferability of tools developed to support the broader research community in (semi)automated data extraction tasks. Collectively, these studies suggest a positive trend toward the development of adaptable, transferable research tools and technologies. However, they also underscore the need for ongoing effort across and between diverse domains to make continued progress toward broader research applications.

### Open source tools

**Figure 10.**
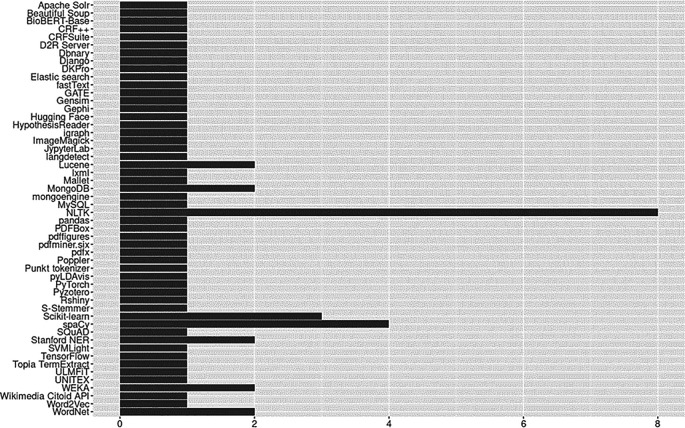
Open source tools.

An outcome we did not anticipate was the substantial number of open source tools, toolkits, and frameworks utilized by our relatively small corpus of articles. Because we were unsure what to expect, we made every effort to capture evidence that might prove useful to social science researchers. We identified 50 different open source technologies including platforms, software, software suites, packages/libraries, algorithms, pre-trained models, controlled vocabularies/thesauri, lexical databases, knowledge representations, and more. Open source tools identified are reported in Figure 10. Of the open source resources available to researchers, the overwhelming majority were Python tools ( *n*=16; see Python Package Index, https://pypi.org/) and 8 of 23 (35%) studies used the Python Natural Language Toolkit (NLTK). The full list of open-source tools and license details are available in the OSF repository (see ‘Underlying Data’ section).

### APA data elements

This section discusses potential for extraction of key data elements of interest, as well as locations (i.e., paper sections), structures, and review tasks addressed by the studies reviewed. We limited this section to reporting tools that users could theoretically access and use to support their own research projects. There were 12 studies that presented systems or artifacts designed to facilitate various tasks associated with identifying and extracting data from published literature. To avoid speculating as to the future availability of these tools, we included all studies which presented tools or systems where authors incorporated user interfaces (UIs), regardless of availability at the time of conducting this base review.

Table 4 provides an overview of data elements targeted as outlined by JARS ( Appelbaum et al., 2018, p. 6). Each tool was assessed for potential to extract specific data elements by manuscript section (i.e., methods and results reporting elements pertinent to meta-analytic research; see Legate & Nimon, 2023b). Where the authors did not state a tool name, we used the description of the tool as presented in the paper (e.g., Bayatmakou et al., 2022; Nayak et al., 2021).

**Table 4.** APA data elements.

			CORWA	CIRRA	DASyR (IR)	Holistic Modifiable Literature Reviewer	Hypothesis Reader	Interactive Text Summarization System	MetaSeer.STEM	OATS	Research Spotlight	Semi-automatic Data Extraction System	SysRev	TableSeer
Manuscript Section	Item	Example Reporting Elements	Li et al. (2022)	Angrosh et al. (2014)	Piroi et al. (2015)	Denzler et al. (2021)	Chen et al. (2021)	Bayatmakou et al. (2022)	Neppalli et al. (2016)	Chen et al. (2020)	Pertsas & Constantopoulos (2018)	Nayak et al. (2021)	Bozada et al. (2021)	Liu et al. (2007)
Methods	Criteria, Data Collection & Participants	Participant selection [setting(s), location(s), date(s), % approached vs. participated], Major/topic-specific demographics [age, sex, ethnicity, achievement level(s), tenure]		✓	✓	✓	✓	✓	✓	✓	✓	✓	✓	✓
	Sample Size, Power & Precision	Intended vs. actual sample size, Sample size determination [power analysis, parameter estimates]	✓	✓	✓	✓	✓	✓	✓	✓	✓	✓	✓	✓
	Measures & Instrumentation	Measures [primary, secondary, covariates], Psychometric properties [reliability coefficients, internal consistency reliability, discriminant/convergent validity, test-retest coefficients, time lag intervals]	✓	✓	✓	✓	✓	✓	✓	✓	✓	✓	✓	✓
	Conditions & Design	Experimental/manipulated [randomized, nonrandomized], Nonexperimental [observational, single- or multi-group], Other [longitudinal, N-of-1, replication]		✓	✓	✓	✓	✓	✓	✓	✓	✓	✓	✓
	Data Diagnostics	Post-collection inclusion/exclusion criteria [criteria to infer missing data, processing of outliers, data distributions, data transformations]		✓	✓	✓	✓	✓	✓	✓	✓	✓	✓	✓
	Analytic Strategy & Hypotheses	Strategy for inferential statistics, Protection against error, Hypothesis (es) [primary, secondary, exploratory]		✓	✓	✓	✓	✓	✓	✓	✓	✓	✓	✓
Results	Participants & Recruitment	Participants (by group/stage), Dates [recruitment, repeated measures]		✓	✓	✓		✓	✓	✓	✓	✓	✓	
	Statistics & Data Analysis	Statistical/data-analytic methods, Missing data (frequency or %), Missing data methods [MCAR, MAR, MNAR], Validity issues [assumptions, distributions]		✓	✓	✓		✓	✓	✓	✓	✓	✓	
	Complex Analyses	Analytic approach [SEM, HLM, factor analysis], Model details [fit indices], Software, Estimation Technique, Estimation Issues [e.g., convergence]	✓	✓	✓	✓		✓	✓	✓	✓	✓	✓	✓
	Descriptive & Inferential Statistics	Descriptive [total sample size, sample size subgroups/cases, means, standard deviations, correlation matrices], Inferential [ *p*-values, degrees of freedom, mean square effects/errors, effect size estimates, confidence intervals]			✓	✓		✓	✓	✓	✓	✓	✓	✓

Unlike ongoing research that focuses on data extraction from clinical literature (e.g., PICO elements/RCTs; see Schmidt et al., 2023), specific reporting guidelines were not a primary focus of the studies we identified. However, authors described target entities and/or research methods of interest with high levels of specificity. For instance, extracting descriptive statistics, sample size, and Likert scale points ( Neppalli et al., 2016) and extracting research hypotheses from published literature in organizational sciences ( Chen et al., 2021). Despite the lack of discourse surrounding specific reporting guidelines, many of the tools reviewed incorporated some form of user-prompted, annotation- or query-based approach to (semi)automated data extraction. Thus, the collective body of work lends optimism surrounding customizable state-of-the art methods that can support extraction for a wide range of disciplines, research designs, and entities or data elements of interest to social science researchers.

One example of a highly flexible approach is extractive question-answering based on pre-trained Transformer models. Extractive question-answering models are able to generate direct answers from knowledge base in response to natural language questions posed by users ( Kwiatkowski et al. 2019). These tools typically offer enhanced flexibility through user-defined prompts and mechanisms for interactive query refinement. Example tools that incorporated question answering techniques included CIRRA ( Piroi et al., 2015), the Interactive Text Summarization System for Scientific Documents ( Bayatmakou et al., 2022), and OATS ( Chen et al., 2021).

Other types of flexible systems allow users to view excerpts related to specific keywords or queries, supporting expedited identification and labeling of target data elements. For example several tools supported user labeling of data, followed by predictive classification based on user annotations. Although these tools do not automatically extract data for users, they do augment human effort by (semi)automating time consuming tasks associated with data annotation and extraction. For instance, Sysrev ( Bozada et al., 2021) supports researchers in labeling and extracting data by leveraging active learning models developed to replicate user decisions across various review tasks. Likewise, MetaSeer ( Neppalli et al., 2016) developed ML techniques to identify and extract numbers from documents, which were then presented to users for manual annotation. Unlike question-answering models, human-computer interactions in these examples are not based on natural language queries, however, human expertise can be used to ‘train’ ML models to predict future annotation decisions. Similarly, to overcome the time-constraints of open-ended annotation in fields that lack domain-specific dictionaries, DASyR ( Piroi et al., 2015) utilized a combination of user annotations, classification models, and contextual information for populating ontologies. They reported substantial reduction in annotation time, stating that through the DASyR UI “five experts added approximately 30,000 annotations at a speed of 4s/annotation” (p. 595).

Lastly, we note the utility of NER for the advancement of (semi)automated extraction of APA defined data elements. NER methodologies can be leveraged alongside classification models ( Nayak et al., 2021), linked to domain specific ontologies or other knowledge bases ( Piroi et al., 2015), or incorporated as stand-alone modules integrated into larger modifiable frameworks ( Denzler et al. 2021). In addition to pre-trained NER models for identification and extraction of named entities, Research Spotlight ( Pertsas & Constantopoulos, 2018) also exploited lexico-syntactic patterns in the scholarly ontology to identify and extract non-named entities. The Semi-automatic Data Extraction System for Heterogeneous Data Sources ( Nayak et al., 2021) combined features of NER and rule-based chunking to identify and extract phrases on regular expressions as well as named entities contained in the documents. Further, NER can be implemented through open source tools as demonstrated by Denzler et al. (2021) and Nayak et al. (2021).

### Structure, location, and review tasks

Table 5 provides an overview of structure and location of extracted data elements, followed by review tasks supported by tools identified. The majority developed approaches for (semi)automating extraction of data from any section of full text research articles. Two studies tested the proposed techniques on specific article sections, including titles and abstracts ( Bayatmakou et al., 2022) and introduction and background sections ( Li et al., 2022). Regarding structure from which data were extracted, all except one extracted from unstructured text, two extracted from both tabular structures (i.e., tables) and text ( Nayak et al., 2021; Pertsas & Constantopoulos, 2018), and one was designed specifically to extract elements from tables (TableSeer; Liu et al., 2007).

**Table 5.** Structure, location, review tasks.

Category	Item	CORWA	CIRRA	DASyR (IR)	Holistic Modifiable Literature Reviewer	Hypothesis Reader	Interactive Text Summarization System	MetaSeer.STEM	OATS	Research Spotlight	Semi-automatic Data Extraction System	SysRev	TableSeer
		Li et al. (2022)	Angrosh et al. (2014)	Piroi et al. (2015)	Denzler et al. (2021)	Chen et al. (2021)	Bayatmakou et al. (2022)	Neppalli et al. (2016)	Chen et al. (2020)	Pertsas & Constantopoulos (2018)	Nayak et al. (2021)	Bozada et al. (2021)	Liu et al. (2007)
Structure	Extract from Text	✓	✓	✓	✓	✓	✓	✓	✓	✓	✓	✓	
	Extract from Tables									✓	✓		✓
Location	Title & Abstract		✓	✓	✓	✓	✓	✓	✓	✓	✓	✓	✓
	Introduction & Background	✓	✓	✓	✓	✓	✓	✓	✓	✓	✓	✓	✓
	Methods		✓	✓	✓	✓	✓	✓	✓	✓	✓	✓	✓
	Results		✓	✓	✓	✓	✓	✓	✓	✓	✓	✓	✓
	Discussion		✓	✓	✓	✓	✓	✓	✓	✓	✓	✓	✓
Review Tasks	1 Formulate Review Question												
	2 Find Previous Reviews				✓							✓	
	3 Write Protocol												
	4 Devise Search Strategy												
	5 Search				✓		✓		✓	✓		✓	✓
	6 De-duplicate				✓								
	7 Screen Abstracts				✓		✓					✓	
	8 Obtain Full Text									✓			
	9 Screen Full Text											✓	
	10 Snowball				✓								
	11 Extract Data	✓	✓	✓	✓	✓	✓	✓	✓	✓	✓	✓	✓
	12 Synthesize Data	✓			✓				✓		✓	✓	
	13 Re-check literature												
	14 Meta-Analyze												
	15 Write up review												

All tools focused heavily on tasks related to data extraction (e.g., identification, labeling/annotation, ontology population), which was anticipated based on our search strategy and inclusion criteria. However, several studies advanced solutions for supporting other SLR tasks or stages (see Tsafnat et al., 2014). The most common task (excluding data extraction) was literature search ( Bayatmakou et al., 2022; Bozada et al., 2021; Chen et al., 2020; Denzler et al., 2021; Liu et al., 2007; Pertsas & Constantopoulos, 2018). Many tasks listed are likely supported by a range of computational tools and techniques (e.g., synthesize and meta-analyze results); readers interested in (semi)automating other SLR stages are referred to the Systematic Review Toolbox for an extensive catalogue of tools and methods ( Marshall et al., 2022).

### Challenges

Throughout the corpus a number of challenges were presented. These challenges included difficulties in identifying functional structures within unstructured texts ( Shen et al., 2022), extracting data from PDF file sources ( Nayak et al., 2021; Goldfarb-Tarrant et al., 2020; Iwatsuki et al., 2017), and accurately detecting in-line mathematical expressions ( Iwatsuki et al., 2017). Computational complexity created another significant obstacle for researchers, with issues arising from text vectorization methods, optimization problems, and the computational resources required by neural network frameworks ( Bayatmakou et al., 2022; Anisienia et al., 2021). Furthermore, challenges associated with annotation, particularly biases introduced through the automated processes and limitations of available datasets, were a topic of discourse ( Li et al., 2022; Nowak & Kunstman, 2019; Torres et al., 2012).

Compared to the medical field, domain-specific challenges, particularly those in social sciences and related fields, necessitated tailored approaches, which can become time-consuming as researchers often lack sufficient training data to develop robust classifiers ( Chen et al., 2021; Aumiller et al., 2020; Zielinski & Mutschke, 2017). Additionally, meta-analytic methods often face hurdles related to data representation variability, which has significant limitations in the use of data extraction tools, and class imbalance in the development of classification tasks ( Aumiller et al., 2020; Neppalli et al., 2016; Goldfarb-Tarrant et al., 2020).

## Conclusions

The findings of the baseline review indicate that when considering the process of automating systematic review and meta-analysis information extraction, social science research falls short as compared to clinical research that focuses on automatic processing of information related to the PICO framework (i.e., Population, Intervention, Control, and Outcome; Eriksenand Frandsen, 2018; Tsafnat et al., 2014). For example, while an LSR focusing on clinical research that is based on the PICO framework yielded 53 studies that included original data extraction ( Schmidt et al., 2021), the present review of social science research yielded only 23 relevant studies. This is not necessarily surprising when considering the breadth of social science research and the lack of unifying frameworks and domain specific ontologies ( Göpfert et al., 2022; Wagner et al., 2022).

With a few exceptions, most tools we identified were either in the infancy stage and not accessible to applied researchers, were domain specific, or require substantial manual coding of articles before automation can occur. Additionally, few solutions considered extraction of data from tables, which is where many elements (e.g., effect sizes) reside that social and behavioral scientists analyze. Further, development appears to have ceased for many of the tools identified.

We found no evidence indicating hesitation on the part of social science researchers to adopt data extraction tools, on the contrary, abstractive text summarization approaches continue to gain traction across social science domains ( Cairo et al., 2019; Feng et al., 2017). While these methods aid researchers in distilling complex information into meaningful insights, there remains a gap in technologies developed to augment human capabilities in the extraction of key data entities of interest for secondary data collection from quantitative empirical reports.

The impact of time-intensive research activities on translational value is not a new concern for the SLR research community. In many social sciences, emphasis is often placed on practical application and translational value, underscoring the importance of efficient research methodologies ( Githens, 2015). Further development of the identified systems and techniques could mitigate time delays that often result in outdated information as researchers cannot feasibly include all new evidence that may be released throughout the lifetime of a given project ( Marshall & Wallace, 2019).

### Limitations

As with any method that involves subjectivity, results can be influenced by a variety of factors (e.g., study design, publication bias, researcher judgment, etc.). We worked diligently to conduct this review and document our procedures in a systematic and transparent manner; however, efforts to replicate our search strategy or screening processes may not result in the same corpus or reach the same conclusions ( Belur et al., 2018). This baseline review presented an opportunity to better develop our search and screening strategy, methodological procedures, and research goals. Moving forward, we have developed a codebook and assessment procedures to increase the transparency and reliability of our research.

A second limitation of this study was the omission of snowballing as a search strategy. Though we did not uncover applied secondary research articles utilizing automation tools, several potentially useable tools and systems were discovered in the course of this review. For future iterations of this LSR, we plan to incorporate forward snowballing from relevant articles in previous searches as part of our formalized search strategy (see Wohlin et al., 2022).

Finally, in this baseline review, we did not capture techniques used for optimization, training, or fine-tuning on specific datasets or tasks. Several techniques surfaced while conducting this review, such as class modifiers (e.g., OvR; Aumiller et al., 2020), genetic algorithms ( Bayatmakou et al., 2022; Torres et al. (2012), Adam optimizer ( Nowak & Kunstman, 2019); Shen et al., 2022), cross entropy loss ( Chen et al., 2020; Li et al., 2022), Universal Language Model Fine-tuning (e.g., ULMFiT; Anisienia et al., 2021), and back-propagation optimizers ( Chen et al., 2020; Anisienia et al., 2021). With increasing applications of pre-trained language models that can be fine-tuned for specific applications ( Jurafsky & Martin, 2024), inclusion of training and optimization approaches would provide a more comprehensive framework for reporting findings on ML/NLP approaches to data extraction. We plan to supplement future iterations of this review by capturing various optimization and training methods.

## Ethics and consent

Ethical approval and consent were not required.

## Data Availability

OSF: (Semi)Automated Approaches to Data Extraction for Systematic Reviews and Meta-Analyses in Social Sciences: A Living Review ( Legate & Nimon, 2024). Open Science Framework: https://doi.org/10.17605/OSF.IO/C7NSA ( Legate & Nimon, 2024). This project contains the following underlying data:
• Baseline Review Underlying Data
○ Baseline Review Results.xlsx ○ Baseline Search Results Folder (a folder containing results by each search source) ○ Open Source Tools.xlsx
• Baseline Review Extended Data
○ Baseline Review Deduplication and Screening.docx ○ Baseline Review Search Strategy.docx ○ Baseline Review PRISMA Checklist.docx ○ LSR Codebook.docx ○ Regex to Boolean Sytnax.xlsx
• Baseline Review Code
○ Adapted code files and results for automated search and screening for ACL, ArXIV, and DBLP full database dumps. Data are available under the terms of the Creative Commons Attribution 4.0 International Public License (CC-BY 4.0). Open Science Framework: https://doi.org/10.17605/OSF.IO/C7NSA( Legate & Nimon, 2023a). This project contains the following extended data:
• Extraction Techniques Revised.docx – categories and descriptions of data extraction techniques, architecture components, and evaluation metrics of interest • Review Classifications.docx – review tasks and stages of interest • Target Data Elements.docx – key elements of interest for targeted data elements • Comprehensive List of Eligible Data Elements.xlsx – comprehensive list of elements with extraction potential per APA JARS • Search Strategy.docx – search syntax for preliminary search in Web of Science • APA & Cochrane Data Elements.xlsx – tabled data elements for Cochrane reviews, APA Module C (clinical trials), and APA (all study designs) Data are available under the terms of the Creative Commons Attribution 4.0 International Public License (CC-BY 4.0). This study follows PRISMA reporting guidelines ( Page et al., 2021). Open Science Framework: PRISMA checklist for ‘Open Science Framework: (Semi)Automated Approaches to Data Extraction for Systematic Reviews and Meta-Analyses in Social Sciences: A Living Review’. https://doi.org/10.17605/OSF.IO/C7NSA ( Legate & Nimon, 2024). Data are available under the terms of the Creative Commons Attribution 4.0 International Public License (CC-BY 4.0).

## Software availability

• Source code available from: https://github.com/mcguinlu/COVID_suicide_living
• Archived source code: http://doi.org/10.5281/zenodo.3871366 ( McGuinness & Schmidt, 2020).
• The adapted version of the source code for automated searching: https://doi.org/10.17605/OSF.IO/C7NSA
• Archived source code: https://doi.org/10.17605/OSF.IO/C7NSA ( Legate & Nimon, 2024).
• License: MIT
