# Supplementary material for: (Semi)automated approaches to data extraction for systematic reviews and meta-analyses in social sciences: A living review
Source: F1000Res. 2024 Sep 26;13:664. Originally published 2024 Jun 20. [Version 2] doi: 10.12688/f1000research.151493.2 (PMC11364972; doi:10.12688/f1000research.151493.2)
Supplement: Peer review history

# Reviewer report: Reviewer response for version 2

**DOI:** 10.5256/f1000research.172337.r349101

**Published:** 2025-01-03

**Authors:** Joshua Polanin

**Affiliations:** 1 American Institutes for Research, Arlington, Virginia, USA

Thank you for the opportunity to submit this peer-review. I found the manuscript engaging and timely. I have several issues the authors can address ahead of final submission. 

 1. I truly appreciated the lit review of related efforts. However, the organization of the section made it difficult for me to follow what had been done and what was ongoing, and importantly, where the other work potentially overlaps with this manuscript's work. I'm not sure exactly how the authors should restructure this section, but I would strongly urge the authors to consider it. 

 2. Please make more clear that this manuscript is only looking at evaluations of tools/models/applications, and not a roundup of all the available AI tools. I agree that the manuscript's framing is useful, but it took me a while to understand that the authors were only interested in that aspect. 

 3. I thought the methods section is pretty good and clear. 

 4. The results are useful and well organized. Some of the figures are difficult to read and could use something beyond the base ggplot design. Shading or color or plots go a long way. 

 5. I think the authors should re-examine their Conclusions section and really try and outline main findings in a really clear way. Right now it's tough to tell what they are. Relatedly, I think there's more limitations here than what is listed. This again goes back to the scope, but I think readers who zoom over the lit review will miss that this manuscript is only interested in evaluations of current tools. This means that many applications making claims about their usefulness (i.e., Elicit and other products) have not been included. Especially given the emphasis on qualitative summaries, the authors need to make clear that those tools have *not* been evaluated in the types of ways that the tools mentioned this article have. 

 6. I appreciated the transparency and reporting done. Nice work!

 Overall this is a great article and will make a strong contribution. But a bit more could be done to clarify. I wish the authors good luck in finalizing!

Are the rationale for, and objectives of, the Systematic Review clearly stated?

Yes

Is the statistical analysis and its interpretation appropriate?

Yes

If this is a Living Systematic Review, is the ‘living’ method appropriate and is the search schedule clearly defined and justified? (‘Living Systematic Review’ or a variation of this term should be included in the title.)

Yes

Are sufficient details of the methods and analysis provided to allow replication by others?

Partly

Are the conclusions drawn adequately supported by the results presented in the review?

Partly

Reviewer Expertise:

I'm an expert in research synthesis methods, designing applications for conducting syntheses. I've worked on both AI-based and non-AI-based architectures.

I confirm that I have read this submission and believe that I have an appropriate level of expertise to confirm that it is of an acceptable scientific standard, however I have significant reservations, as outlined above.

# Reviewer report: Reviewer response for version 1

**DOI:** 10.5256/f1000research.166140.r298395

**Published:** 2024-08-30

**Authors:** Lena Schmidt

**Affiliations:** 1 National Institute for Health and Care Research Innovation Observatory, Population Health Sciences Institute, Newcastle University, Newcastle upon Tyne, England, UK

Thank you for providing this interesting review about automated data extraction from social science studies and model architectures, evaluation, data processing and more. Especially the information in tables, such as tables 3, 4, and 5 are adding a lot of value. I am not an expert in social science studies but am familiar with the general field of automated data extraction. Therefore I have just some very minor comments and questions for clarification below:

 The reference from Yu et al. below is mostly concerning screening automation and not data extraction if I am not missing a major point in the paper. If that is correct then there may exist better works to reference in this context? “the process of extracting data from primary research is a labor-intensive effort, fraught with the potential for human error (see Pigott & Polanin, 2020; Yu et al., 2018).” I am not an expert in social science research, but a few included references in Table 2 caught my eye. For example Iwatsuki et al. (2017) about detecting in-line mathematical expressions or Torres et al. (2012) about software engineering or later Nayak et al. (2021) about cotton industry? Regarding this sentence in the conclusions, it might be more up-to-date to reference the review update from 2023 with 76 included papers: “For example, while an LSR focusing on clinical research that is based on the PICO framework yielded 53 studies that included original data extraction (Schmidt et al., 2021)” One of the challenges with living updates is to adapt the search whenever there are new developments in a field of research. You may have already considered adapting the search strategy to make sure that new methods relying on large language models (LLM) like GPT or T5 are picked up? There may be relevant articles coming through soon, for example https://arxiv.org/abs/2405.14445  may be of interest for a future review update as it looks at social science study data extraction and if it is, then it would be good to make sure that the search can pick up the terminology correctly. In the methodology section, could you please state the dates when the search relevant to the baseline review cutoff was conducted (for each data source if different) ?"

Are the rationale for, and objectives of, the Systematic Review clearly stated?

Yes

Is the statistical analysis and its interpretation appropriate?

Yes

If this is a Living Systematic Review, is the ‘living’ method appropriate and is the search schedule clearly defined and justified? (‘Living Systematic Review’ or a variation of this term should be included in the title.)

Yes

Are sufficient details of the methods and analysis provided to allow replication by others?

Yes

Are the conclusions drawn adequately supported by the results presented in the review?

Yes

Reviewer Expertise:

Systematic review automation, automated data extraction (clinical trials), natural language processing, living reviews

I confirm that I have read this submission and believe that I have an appropriate level of expertise to confirm that it is of an acceptable scientific standard.

# Response

**Published:** 2024-09-22

**Authors:** Amanda Legate

**Affiliations:** University of Texas Tyler

We are honored that you agreed to review our research and sincerely appreciate your thoughtful review and feedback. Please find responses to each comment below.

 Comment 1

 The reference from Yu et al. below is mostly concerning screening automation and not data extraction if I am not missing a major point in the paper. If that is correct then there may exist better works to reference in this context? “the process of extracting data from primary research is a labor-intensive effort, fraught with the potential for human error (see Pigott & Polanin, 2020; Yu et al., 2018).”

 Comment 1: Response

 Thank you for catching this oversight. You are absolutely correct; Yu et al. (2018) primarily focused on screening automation for primary study selection rather than data extraction stages of SLRs. We have removed this reference from the sentence to align with the context.

 Comment 2

 I am not an expert in social science research, but a few included references in Table 2 caught my eye. For example Iwatsuki et al. (2017) about detecting in-line mathematical expressions or Torres et al. (2012) about software engineering or later Nayak et al. (2021) about cotton industry?

 Comment 2: Response

 Thank you for your insights on the relevance of references in Table 2. Our search strategy was intentionally broad to include studies utilizing (semi)automated data extraction methods across various domains, provided they were not solely focused on clinical research. The goal was to ensure comprehensiveness; however, we understand your concern regarding the ambiguity of some references' relevance to social sciences. As our study is a "living" review, we see this as an excellent opportunity to consider refining our inclusion criteria in future updates. We will explore more targeted approaches that can help streamline the search strategy, potentially focusing on research that more directly applies to social sciences or explicitly demonstrates transferable methodologies that align with the needs of social science researchers. Additionally, we are discussing options for collaborating with experts who specialize in bibliometric analysis or search strategy optimization to ensure that our review remains focused, relevant, and complementary to your work.

 Comment 3

 Regarding this sentence in the conclusions, it might be more up-to-date to reference the review update from 2023 with 76 included papers: “For example, while an LSR focusing on clinical research that is based on the PICO framework yielded 53 studies that included original data extraction (Schmidt et al., 2021)”.

 Comment 3: Response

 Thank you for pointing out this important update, we appreciate your diligence in ensuring that references are current and reflective of the most recent findings. The manuscript has been revised to reference the 2023 update of your LSR to accurately reflect the most up-to-date results.

 Comment 4

 One of the challenges with living updates is to adapt the search whenever there are new developments in a field of research. You may have already considered adapting the search strategy to make sure that new methods relying on large language models (LLM) like GPT or T5 are picked up? There may be relevant articles coming through soon, for example https://arxiv.org/abs/2405.14445  may be of interest for a future review update as it looks at social science study data extraction and if it is, then it would be good to make sure that the search can pick up the terminology correctly.

 Comment 4: Response

 Thank you for highlighting the importance of adapting the search strategy to capture emerging developments in automation technologies, particularly those involving large language models (LLMs) like GPT or T5. We completely agree that a key aspect of maintaining the relevance and rigor of a living systematic review is to continuously update the search strategy to reflect the current state-of-the-art in the field. We will incorporate this valuable feedback into future iterations by updating our search terms and strategies to include LLM-related methodologies and terminologies, ensuring the inclusion of new and relevant articles. The paper you referenced ( https://arxiv.org/abs/2405.14445) serves as an excellent example, and we will use it to refine our search criteria. This approach will help us stay current with advances in data extraction techniques. Thank you for providing specific references to guide this adaptation.

 Comment 5

 In the methodology section, could you please state the dates when the search relevant to the baseline review cutoff was conducted (for each data source if different) ?"

 Comment 5: Response

 Thank you for this thoughtful suggestion. While we reported the search dates in the extended data files housed in OSF, we agree with you that including them directly in the Methods section would add clarity and value for readers. We have updated the section to specify the dates when searches were conducted for each data source, ensuring this information is clear and accessible to readers.

# Reviewer report: Reviewer response for version 1

**DOI:** 10.5256/f1000research.166140.r298402

**Published:** 2024-08-19

**Authors:** Biljana Macura

**Affiliations:** 1 Stockholm Environment Institute, Stockholm, Sweden

This manuscript represents an important contribution to the evidence synthesis methodology. Given the rise of AI technology, a living evidence base on approaches to data extraction will be very useful. However, the manuscript could benefit from improved clarity. Below are my comments:

1. Title:
  1. Clarify the type of data being extracted (qualitative, quantitative, or mixed).
  2. Since this review does not include any qualitative or quantitative synthesis per se, but rather provides an overview of the field (methods for semi-automated data extraction), I suggest removing "living systematic review" and adding "living systematic map."
2. Abstract:
  1. The summary of methods could include more detailed information on searches, screening, critical appraisal, and synthesis. Please specify which standards for review conduct were followed.
  2. The summary of results could provide more information (briefly) about the included studies.
3. Keywords:
  1. Avoid repeating terms already present in the title
4. Introduction:
  1. The focus of this review—extraction tools for quantitative data—should be more explicitly stated. This emphasis needs to be clearer in the introduction and reflected in the title, as mentioned earlier. Specifically, the first paragraph of the Introduction should be revised to concentrate on the review topic—quantitative data extraction and existing tools—rather than a general introduction to meta-science or related areas.
  2. Additional details are needed on how this review contributes to and complements existing reviews on the topic. This information should be included in the "Related Research" section.
5. Objectives:
  1. It would be helpful to define what is included under *“social science research domains”.*
6. Methods:
  1. Authors should be transparent and explicit about the guidelines and standards for both conduct and reporting that were used. Please clarify this at the beginig of the Methods section.
  2. The methods section should begin by addressing any deviations from the protocol. If there were no deviations, this should be clearly stated as well.
  3. Did you use any automation technologies to screen or select studies for this review? If yes, please clarify.
7. Methods/Eligibility criteria:
  1. The eligibility criteria should be explicit about the field within which methods for (semi)automated data extraction are applied.
  2. A definition of “(semi)automated” is needed. The eligibility criteria currently state that semi-automated approaches will be eligible but then refer to “any automated approach to data extraction” in the next sentence. This needs to be clarified—are the focus and criteria on semi-automated or automated approaches? Be more explicit and precise in the description of the eligibility criteria, and ensure alignment with the protocol.
  3. Instead of “We excluded studies labeled as editorials, briefs..” you may write “Editorials, briefs, …were not considered eligible”  (and similar changes may be applied to the following sentence)
8. Methods/ Searches
  1. Be explicit about the citation indices included in your Web of Science subscription and note which library was used to access WoS. This will increase transparency and replicability of your searches.
  2. Clarify why following Schmidt et al.'s search strategy was important, given the different scope of this review. Consider including more social science databases to ensure comprehensive coverage. Did you include the Social Science Citation Index (within WoS)?
  3. Provide explanations for all abbreviations (IEEE, ACL, etc.) in the text.
9. Methods/Study selection
  1. Clarify if three researchers simultaneously screened titles and abstracts (TA), and whether inter-rater reliability (IRR) was calculated for TA screening. How you trained reviewers to apply eligibility criteria?
  2. The sentence *,* *“* *In cases where level of abstraction and potential for transferability could not be determined from the abstract alone, full text articles were reviewed and discussed by all three researchers until consensus was reached*”, should more clearly state that there was NO full-text screening of all records (if this is correct), only of a sub-sample where abstracts did not clearly describe AI technology, etc.  
  3. Relatedly, Figure 1 should be adjusted to avoid giving the false impression that all records were screened in full text.
  4. This review seems to involve META-data extraction rather than DATA extraction. Please adjust the text and figures accordingly.
  5. It is not clear if IRR assessments were conducted for meta-data extraction. Please clarify/be explicit. If IRR was not done, describe how researchers were trained to use the extraction form.
  6. The sentence, “ *coding forms allowed for input of “other” responses (e.g., APA data elements) that were not included in extant reviews that focus on medical and clinical data extraction (e.g., PICO elements)*” is unclear.  Consider removing or clarifying and linking it better with the rest of the text.
  7. Describe the procedure for screening and meta-data extraction of studies authored by the review team.
10. Methods/Critical appraisal and Synthesis
  1. These sections are missing. Please state clearly if a critical appraisal of included studies was conducted and if so, how was it performed. Also, describe how synthesis was conducted.
11. Results/Challenges
  1. Clarify that the described challenges reflect issues within the body of evidence included in this (baseline) review (otherwise this section can be mixed up with review limitations).
12. Conclusions/Limitations
  1. Organize limitations into those related to the methodology used and those related to the evidence base.
  2. Discuss limitations related to the focus on publications in English, the inexhaustive list of search sources, and the lack of grey literature.

Are the rationale for, and objectives of, the Systematic Review clearly stated?

Partly

Is the statistical analysis and its interpretation appropriate?

Not applicable

If this is a Living Systematic Review, is the ‘living’ method appropriate and is the search schedule clearly defined and justified? (‘Living Systematic Review’ or a variation of this term should be included in the title.)

Partly

Are sufficient details of the methods and analysis provided to allow replication by others?

Partly

Are the conclusions drawn adequately supported by the results presented in the review?

Yes

Reviewer Expertise:

Systematic Evidence Synthesis Methodology

I confirm that I have read this submission and believe that I have an appropriate level of expertise to confirm that it is of an acceptable scientific standard, however I have significant reservations, as outlined above.

# Response

**Published:** 2024-09-22

**Authors:** Amanda Legate

**Affiliations:** University of Texas Tyler

Dear Dr. Macura,

 Thank you for your thoughtful and detailed feedback on our manuscript. We appreciate the time and effort you have invested in providing suggestions to enhance our work. We also value rigorous research methods and reporting transparency and would like to clarify several points regarding the reporting guidelines we adhered to and the journal's policies and requirements.

 Our manuscript follows the PRISMA (Preferred Reporting Items for Systematic Reviews and Meta-Analyses) guidelines. As noted in the F1000Research “Article Standards of Reporting” (https://f1000research.com/about/policies#stofrep), systematic reviews published in this journal must adhere to PRISMA guidelines. We have ensured that our reporting aligns with PRISMA's emphasis on transparency, replicability, and comprehensiveness.

 We would like to express our genuine appreciation for the important work you and your colleagues have done in developing the ROSES (Reporting standards for Systematic Evidence Syntheses) guidelines for systematic evidence synthesis in environmental science. Improving transparency and standardization in research reporting is a goal we fully support. While we acknowledge the value of the ROSES guidelines, they were not the reporting standard required or appropriate for our systematic review. We noticed that many of your comments seem to assess our manuscript against the ROSES guidelines (Haddaway et al., 2017a; 2017b; 2018; Haddaway & Macura, 2018). For example, the suggestion to emphasize "meta-data extraction" aligns more with ROSES, whereas PRISMA does not require such differentiation and focuses on clarity in describing the data collection process, whether it involves meta-data or primary data points.

 We believe it is essential to assess our work based on the scope and framework provided by PRISMA rather than extend it beyond its current focus to fit an alternative reporting framework. We are committed to making revisions that enhance the clarity and rigor of our research while remaining consistent with the standards required by the journal.

 Thank you again for your constructive feedback and for considering our clarifications. 

**References**

 Haddaway, N. R., & **Macura, B.** (2018). The role of reporting standards in producing robust literature reviews. *Nature Climate Change, 8*(6), 444–447. https://doi.org/10.1038/s41558-018-0180-3

 Haddaway, N. R., **Macura, B.**, Whaley, P., & Pullin, A. S. (2017a). *ROSES for systematic map reports *(Version 1.0) [Data file]. https://doi.org/10.6084/m9.figshare.5897299 

 Haddaway, N. R., **Macura, B.**, Whaley, P., & Pullin, A. S. (2017b). *ROSES for systematic review reports *(Version 1.0) [Data file]. https://doi.org/10.6084/m9.figshare.5897272

 Haddaway, N. R., **Macura, B.**, Whaley, P., & Pullin, A. S. (2018). ROSES reporting standards for systematic evidence syntheses: Pro forma, flow-diagram and descriptive summary of the plan and conduct of environmental systematic reviews and systematic maps. *Environmental Evidence, 7*(7). https://doi.org/10.1186/s13750-018-0121-7

 Comment 1

 [Title] Clarify the type of data being extracted (qualitative, quantitative, or mixed).

 Comment 2

 [Title] Since this review does not include any qualitative or quantitative synthesis per se, but rather provides an overview of the field (methods for semi-automated data extraction), I suggest removing "living systematic review" and adding "living systematic map."

 Comment 1 & 2: Response

 Thank you for these valuable suggestions regarding the title. We have considered (1) specifying the type of data being extracted in the title and (2) changing the title from "living systematic review" to "living systematic map." However, we have retained the original title to ensure consistency with our pre-registered protocol, adhere to PRISMA reporting standards, and comply with F1000Research guidelines.

 Comment 3

 [Abstract] The summary of methods could include more detailed information on searches, screening, critical appraisal, and synthesis. Please specify which standards for review conduct were followed.

 Comment 3: Response

 Thank you for the suggestion to provide more detailed information on searches, screening, critical appraisal, and synthesis in the abstract to better align with ROSES reporting recommendations. To ensure compliance with the journal's requirements, we followed the PRISMA guidelines for a structured summary, which emphasize conciseness in presenting objectives, eligibility criteria, methods, results, and conclusions. While we understand the desire for additional details, we believe the current abstract aligns with these guidelines but will review it again to ensure optimal clarity.

 Comment 4

 [Abstract] The summary of results could provide more information (briefly) about the included studies.

 Comment 4: Response

 Thank you for the recommendation to provide more information about the included studies within the abstract. We will incorporate a brief summary of the included studies' key characteristics and findings in future updates to this review to enhance clarity and completeness.

 Comment 5

 [Keywords] Avoid repeating terms already present in the title

 Comment 5: Response

 Thank you for highlighting ROSES guidance indicating that keywords do not repeat the title but rather provide additional context. Where appropriate, we will revise keywords to avoid redundancy and enhance discoverability.

 Comment 6

 [Introduction] The focus of this review—extraction tools for quantitative data—should be more explicitly stated. This emphasis needs to be clearer in the introduction and reflected in the title, as mentioned earlier. Specifically, the first paragraph of the Introduction should be revised to concentrate on the review topic—quantitative data extraction and existing tools—rather than a general introduction to meta-science or related areas.

 Comment 6: Response

 Thank you for your feedback on clarifying the focus of our review. Our study does not exclusively focus on extraction tools for quantitative data; it encompasses approaches to data extraction for both quantitative and qualitative data elements relevant to evidence synthesis in systematic reviews and meta-analyses within social sciences. To better reflect this broader focus, we have revised the objective section to explicitly state that the review covers data extraction tools for a range of data types. We hope this adjustment will provide clearer insight into the comprehensive scope of our review.

 Comment 7

 [Introduction] Additional details are needed on how this review contributes to and complements existing reviews on the topic. This information should be included in the "Related Research" section.

 Comment 7: Response

 Thank you for this insightful comment. We agree on the importance of clearly situating our review within the existing literature to highlight its unique contributions. Although we did not adhere to ROSES guidelines for explaining the review's relevance to existing literature, we followed PRISMA guidelines in the "Related Literature" section to identify relevant prior reviews and synthesize their focus, findings, and limitations. We will consider ways to enhance this section to better emphasize our review's distinct contributions moving forward.

 Comment 8

 [Objectives] It would be helpful to define what is included under *“social science research domains”.*

 Comment 8: Response

 Thank you for this suggestion. Our pre-registered research protocol and the "Baseline Review Search Strategy" document (available in the project's OSF repository) provide a comprehensive list of over 100 subject categories included under social science research domains, ranging from sociology and political science to interdisciplinary areas such as "Social Sciences Mathematical Methods." To enhance clarity, we have updated the objectives section to include more details and a reference to the extended data file.

 Comment 9

 [Methods] Authors should be transparent and explicit about the guidelines and standards for both conduct and reporting that were used. Please clarify this at the beginning of the Methods section.

 Comment 9: Response

 We acknowledge that the ROSES guidelines recommend transparency in reporting the guidelines and standards for both conduct and reporting at the beginning of the Methods section. However, in accordance with the journal's article guidelines for living systematic reviews (available from: https://f1000research.com/for-authors/article-guidelines/living-systematic-reviews), this information is provided in the "Reporting Guidelines" section. 

 Comment 10

 [Methods] The methods section should begin by addressing any deviations from the protocol. If there were no deviations, this should be clearly stated as well.

 Comment 10: Response

 Thank you for highlighting this important aspect. While we did not adopt the ROSES reporting standards for this research, we recognize their guidance on stating any deviations from the protocol at the beginning of the methods section. We have addressed any deviations in the appropriate sections of the paper, and additional descriptions are provided in the extended data files to ensure transparency and replicability.

 Comment 11

 [Methods] Did you use any automation technologies to screen or select studies for this review? If yes, please clarify.

 Comment 11: Response

 Thank you for your question. The use of automation technologies is detailed in the "Search Sources" and "Study Selection" subsections of the Methods section. Additionally, to ensure transparency and replicability, further details are provided in the "Software Availability" section, as per F1000Research guidelines.

 Comment 12

 [Methods/Eligibility criteria] The eligibility criteria should be explicit about the field within which methods for (semi)automated data extraction are applied.

 Comment 12: Response

 Thank you for this comment. To ensure clarity, we have referenced the extended data files in the text, which provide comprehensive details and a full list of over 100 research fields. These details are openly available in the project repository, as specified in the protocol (please see response to Comment 8).

 Comment 13

 [Methods/Eligibility criteria] A definition of “(semi)automated” is needed. The eligibility criteria currently state that semi-automated approaches will be eligible but then refer to “any automated approach to data extraction” in the next sentence. This needs to be clarified—are the focus and criteria on semi-automated or automated approaches? Be more explicit and precise in the description of the eligibility criteria and ensure alignment with the protocol.

 Comment 13: Response

 Thank you for your suggestion to clarify the phrasing regarding the eligibility criteria. We have revised the description to specify that the focus is on any "technique" applied for extracting data from literature in a semi-automated manner. This adjustment aligns with the study protocol.

 Comment 14

 [Methods/Eligibility criteria] Instead of “We excluded studies labeled as editorials, briefs..” you may write “Editorials, briefs, …were not considered eligible”  (and similar changes may be applied to the following sentence).

 Comment 14: Response

 Thank you for the suggestion. We have revised the text to use passive construction, as recommended. We have also applied similar changes to the following sentence for consistency.

 Comment 15

 [Methods/ Searches] Be explicit about the citation indices included in your Web of Science subscription and note which library was used to access WoS. This will increase transparency and replicability of your searches.

 Comment 15: Response

 Thank you for this suggestion. To avoid redundancy in the manuscript, we have added a statement directing readers to the extended data files, which provide additional detail related to WoS indices and search settings.

 Comment 16

 [Methods/ Searches] Clarify why following Schmidt et al.'s search strategy was important, given the different scope of this review.

 Comment 16: Response

 Thank you for this comment. To clarify, we followed Schmidt et al.'s search strategy to ensure comprehensive coverage of relevant databases and consistency in methodological rigor, which is important even with a different scope. To avoid redundancy, we have added a reference in the manuscript directing readers to the extended data file and research protocol in our open-access repository, where this rationale is explained in detail.

 Comment 17

 [Methods/ Searches] Consider including more social science databases to ensure comprehensive coverage.

 Comment 17: Response

 Thank you for this valuable suggestion. We appreciate the importance of comprehensive coverage and will consider including additional social science databases in future updates to further enhance the scope of our review.

 Comment 18

 [Methods/ Searches] Did you include the Social Science Citation Index (within WoS)?

 Comment 18: Response

 Yes, the Social Science Citation Index within Web of Science was included. We have updated the text to clarify that all editions, settings, and search syntax used are detailed in the extended data files available in the open-access repository.

 Comment 19

 [Methods/ Searches] Provide explanations for all abbreviations (IEEE, ACL, etc.) in the text.

 Comment 19: Response

 Thank you for the suggestion. We have added explanations for all source abbreviations (e.g., IEEE, ACL) in the text to improve clarity for readers.

 Comment 20

 [Methods/ Study selection] Clarify if three researchers simultaneously screened titles and abstracts (TA), and whether inter-rater reliability (IRR) was calculated for TA screening. How you trained reviewers to apply eligibility criteria?

 Comment 20: Response

 Thank you for your question. The "Study Selection" section of the paper details independent screening procedures, training process for reviewers on applying eligibility criteria, and inter-rater reliability (IRR) considerations.

 Comment 21

 [Methods/ Study selection] The sentence *, “In cases where level of abstraction and potential for transferability could not be determined from the abstract alone, full text articles were reviewed and discussed by all three researchers until consensus was reached*”, should more clearly state that there was NO full-text screening of all records (if this is correct), only of a sub-sample where abstracts did not clearly describe AI technology, etc.  

 Comment 21: Response

 Thank you for this observation. While ROSES guidelines provide alternative flowchart formatting and descriptions, we adhered to PRISMA guidelines. According to the PRISMA flowchart (Figure 1), a total of 11,336 records were identified, and after deduplication, 10,644 articles underwent title and abstract screening. As indicated in the flowchart, only 46 articles proceeded to the full-text screening stage, which occurred separately.

 Comment 22

 [Methods/ Study selection] Relatedly, Figure 1 should be adjusted to avoid giving the false impression that all records were screened in full text.

 Comment 22: Response

 Thank you for highlighting this concern. The current figure indicates that 46 articles were included in the full-text screening stage, making it clear that not all records were screened in full text. However, we will consider expanding the flowchart in future updates to provide additional details that could further enhance transparency and clarity.

 Comment 23

 [Methods/ Study selection] This review seems to involve META-data extraction rather than DATA extraction. Please adjust the text and figures accordingly.

 Comment 23: Response

 Thank you for your observation. While ROSES emphasizes distinguishing between meta-data extraction and data extraction, PRISMA does not make this distinction as explicitly. Our paper follows PRISMA guidelines, focusing on transparency and completeness in documenting the tools, databases, and criteria used for extraction.

 Comment 24

 [Methods/ Study selection] It is not clear if IRR assessments were conducted for meta-data extraction. Please clarify/be explicit. If IRR was not done, describe how researchers were trained to use the extraction form.

 Comment 24: Response

 Thank you for raising this point. The "Study Selection" section of the paper details this information and discusses inter-rater reliability (IRR) assessments.

 Comment 25

 [Methods/ Study selection] The sentence, “ *coding forms allowed for input of “other” responses (e.g., APA data elements) that were not included in extant reviews that focus on medical and clinical data extraction (e.g., PICO elements)*” is unclear.  Consider removing or clarifying and linking it better with the rest of the text.

 Comment 25: Response

 Thank you for this suggestion. We have refined the statement to improve clarity and ensure it is better linked with the surrounding text.

 Comment 26

 [Methods/ Study selection] Describe the procedure for screening and meta-data extraction of studies authored by the review team.

 Comment 26: Response

 Thank you for highlighting ROSES guidance surrounding procedures for handling studies authored by the review team. However, no alternative procedures were implemented; therefore, there are no additional procedures to report.

 Comment 27

 [Methods/ Critical appraisal and Synthesis] These sections are missing. Please state clearly if a critical appraisal of included studies was conducted and if so, how was it performed. Also, describe how synthesis was conducted.

 Comment 27: Response

 Thank you for your comment. These sections are specific to ROSES guidelines. These sections are not required by PRISMA or the journal's reporting standards.

 Comment 28

 [Results/Challenges] Clarify that the described challenges reflect issues within the body of evidence included in this (baseline) review (otherwise this section can be mixed up with review limitations).

 Comment 28: Response

 To avoid confusion with review limitations, we have revised the first sentence of this section to clarify that the challenges discussed specifically reflect issues within the body of evidence included in this baseline review.

 Comment 29

 [Conclusions/Limitations] Organize limitations into those related to the methodology used and those related to the evidence base.

 Comment 29: Response

 While our research and protocol were developed following PRISMA guidelines rather than ROSES, which requires a structured discussion of limitations, we appreciate the value of differentiating between methodological constraints and evidence base gaps. We will consider this distinction in future updates to enhance clarity.

 Comment 30

 [Conclusions/Limitations] Discuss limitations related to the focus on publications in English, the inexhaustive list of search sources, and the lack of grey literature.

 Comment 30: Response

 Thank you for the suggestion. We have updated the limitations section to address the focus on publications in English, the inexhaustive list of search sources, and the lack of grey literature.

# Reviewer report: Reviewer response for version 1

**DOI:** 10.5256/f1000research.166140.r298396

**Published:** 2024-07-16

**Authors:** Fred Oswald

**Affiliations:** 1 Rice University, Houston, Texas, USA

Overall, this paper is an excellent review of automated data-extraction methods for the purposes of synthetic reviews and meta-analysis. To my knowledge, there is no such review in the literature, and yet given the rise in AI-based technologies, there is a rising need for researchers to have a single resource identifying these extraction methods. This review nicely summarizes the types of tools that are out there, but it might further tie the tools more closely to a checklist that reflects must be typically must be accomplished when conducting meta-analysis (e.g., identifying literature, extracting sample sizes and effect sizes, converting effect sizes when necessary, coding effect sizes into variables, associated moderators, associated reliability coefficients, dealing with missing data). This would give the reader a better sense of what* gets automated and serves their purposes (e.g., you can take the ‘model architectures and components’ section and populate the checklist/framework with these AI tools/functions) . Also, though it is certainly useful to document when and how humans are compared to automated systems, the level of accuracy reported (e.g., errors of commission and omission by automated systems) would be useful as well (i.e., are these automated systems any good? when systems agree with humans, when are they agreeing in an accurate way vs. a biased way?)

 Thank you for the opportunity to review – again, this will be a valuable paper to readers.

Are the rationale for, and objectives of, the Systematic Review clearly stated?

Yes

Is the statistical analysis and its interpretation appropriate?

Yes

If this is a Living Systematic Review, is the ‘living’ method appropriate and is the search schedule clearly defined and justified? (‘Living Systematic Review’ or a variation of this term should be included in the title.)

Yes

Are sufficient details of the methods and analysis provided to allow replication by others?

Yes

Are the conclusions drawn adequately supported by the results presented in the review?

Yes

Reviewer Expertise:

NA

I confirm that I have read this submission and believe that I have an appropriate level of expertise to confirm that it is of an acceptable scientific standard.

# Response

**Published:** 2024-09-22

**Authors:** Amanda Legate

**Affiliations:** University of Texas Tyler

Comment 1

 Also, though it is certainly useful to document when and how humans are compared to automated systems, the level of accuracy reported (e.g., errors of commission and omission by automated systems) would be useful as well (i.e., are these automated systems any good? when systems agree with humans, when are they agreeing in an accurate way vs. a biased way?)

 Thank you for the opportunity to review – again, this will be a valuable paper to readers.

 Comment 1: Response

 Thank you for this insightful suggestion. We agree that evaluating the accuracy of automated systems compared to human assessments, particularly regarding errors of commission and omission, would provide valuable insights into their effectiveness and potential biases. Understanding when automated systems align with human judgments accurately is indeed crucial for advancing the field.

 Given the "living" nature of our review, we see this as an important focus for future updates. Although additional technical expertise may be required to conduct a comprehensive comparative assessment of these accuracy measures, we hope to expand our team to include experts in areas of data science and AI evaluation. This addition will enhance the rigor of our review and address critical questions surrounding the reliability of automated tools.

 We appreciate your valuable feedback and are committed to integrating these considerations in future versions of our living review.
